# Compartmentalization of soluble endocytic proteins in synaptic vesicle clusters by phase separation

**DOI:** 10.1016/j.isci.2023.106826

**Published:** 2023-05-06

**Authors:** Tomofumi Yoshida, Koh-ichiro Takenaka, Hirokazu Sakamoto, Yusuke Kojima, Takumi Sakano, Koyo Shibayama, Koki Nakamura, Kyoko Hanawa-Suetsugu, Yasunori Mori, Yusuke Hirabayashi, Kenzo Hirose, Shigeo Takamori

**Affiliations:** 1 Laboratory of Neural Membrane Biology, Graduate School of Brain Science, Doshisha University, Kyoto 610-0394, Japan; 2 Department of Pharmacology, Graduate School of Medicine, The University of Tokyo, Tokyo 113-0033, Japan; 3 International Research Center for Neurointelligence (WPI-IRCN), The University of Tokyo, Tokyo 113-0033, Japan; 4 Department of Chemistry and Biotechnology, School of Engineering, The University of Tokyo, Tokyo 113-0033, Japan

**Keywords:** Biological sciences, Molecular biology, Cell biology

## Abstract

Synaptic vesicle (SV) clusters, which reportedly result from synapsin’s capacity to undergo liquid-liquid phase separation (LLPS), constitute the structural basis for neurotransmission. Although these clusters contain various endocytic accessory proteins, how endocytic proteins accumulate in SV clusters remains unknown. Here, we report that endophilin A1 (EndoA1), the endocytic scaffold protein, undergoes LLPS under physiologically relevant concentrations at presynaptic terminals. On heterologous expression, EndoA1 facilitates the formation of synapsin condensates and accumulates in SV-like vesicle clusters via synapsin. Moreover, EndoA1 condensates recruit endocytic proteins such as dynamin 1, amphiphysin, and intersectin 1, none of which are recruited in vesicle clusters by synapsin. In cultured neurons, like synapsin, EndoA1 is compartmentalized in SV clusters through LLPS, exhibiting activity-dependent dispersion/reassembly cycles. Thus, beyond its essential function in SV endocytosis, EndoA1 serves an additional structural function by undergoing LLPS, thereby accumulating various endocytic proteins in dynamic SV clusters in concert with synapsin.

## Highlights

• Endophilin A1 undergoes liquid-liquid phase separation in presynaptic terminals
• EndoA1 condensates accumulate in SV-like clusters in a synapsin-dependent manner
• EndoA1 condensates recruit various endocytic proteins into the condensed phase
• EndoA1-synapsin condensates may maintain dynamic SV clusters with endocytic proteins

## Graphical abstract

**Figure:**
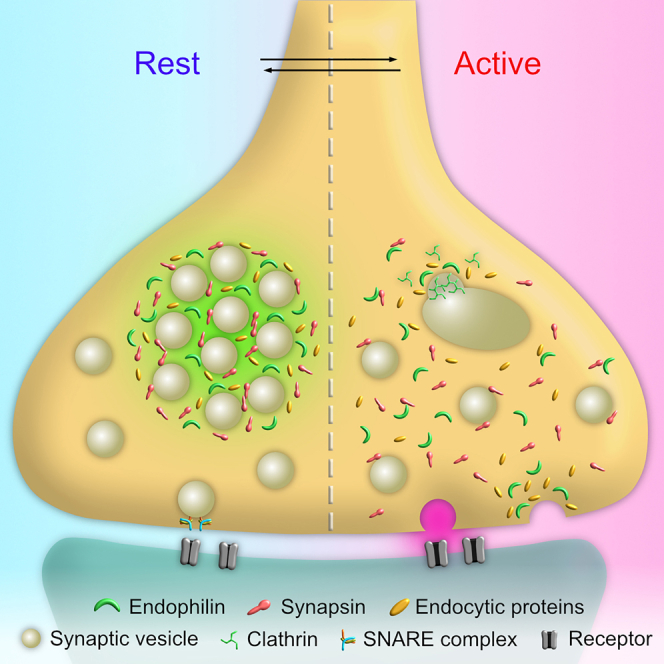

## Introduction

Interneuronal communication occurs primarily at specialized cellular junctions called synapses. On the presynaptic side, synaptic vesicles (SVs), tiny membranous sacs ∼40 nm in diameter that store neurotransmitter molecules, form clusters in the vicinity of release sites called active zones (AZs) and undergo activity-dependent exocytosis, thereby releasing neurotransmitter molecules, which are then recognized by receptors on neighboring postsynaptic cells.^1^ The number of SVs in a presynaptic terminal varies from several hundreds to tens of thousands, depending on the synapse type, but SVs are categorized into three functionally distinct groups: a readily releasable pool (RRP) that undergoes exocytosis immediately on the arrival of an action potential, a recycling pool that replenishes the RRP during sustained stimulation, and a resting pool that participates in exocytosis only on prolonged stimulation.^2^^,^^3^ Because strength and plasticity of synaptic transmission critically depends on the size and availability of SV pools for release, maintenance and faithful reformation of SV clusters, especially during and after intensive stimulation, is essential for proper synaptic transmission.

Recently, the liquid-like nature of synapsin, the abundant soluble protein family specifically expressed in neurons, has been demonstrated *in vitro*, and may explain how this protein helps to form SV clusters.^4^ Synapsin undergoes liquid-liquid phase separation (LLPS) to form micron-sized, membrane-less organelles, and also recruits liposomes mimicking the lipid composition of SVs, which contain negatively charged lipids.^5^ Such properties have been recapitulated in heterologous expression in COS7 cells, in which co-expression of synapsin with synaptophysin, which promotes formation of small vesicles, resulted in formation of SV-like clusters in the cytoplasm.^6^ However, formation of synapsin condensates *in vitro* takes ∼1 h, and mere expression of synapsin in COS7 cells does not result in droplet-like structures.^4^^,^^6^ Although dispersion of SV clusters by injection of an antibody that impedes formation of synapsin LLPS into lamprey synapses suggests that synapsin LLPS predominantly contributes to maintenance of SV clusters also in living synapses,^7^ it remains controversial whether additional components are necessary to form SV clusters at rest, and also for reassembly of clusters during and after intensive stimulation. Of interest, α-synuclein, another SV-associated soluble protein, also has potential to undergo LLPS. It also aids formation of synapsin condensates in heterologous cells, and modulates SV clusters in a manner very similar to synapsin,^8^ raising the possibility that other presynaptic proteins can modulate SV clusters formed by synapsin.

In addition to synapsin, a group of endocytic proteins, including intersectin, amphiphysin, dynamin, epsin synaptojanin, endophilin, and Eps15, are also highly enriched in SV clusters, and undergo activity-dependent redistribution cycles between SV clusters and peri-active zones in lamprey synapses,^9^ in *C*. *elegans*^10^ and in mice.^11^ Notably, these endocytic proteins often contain one or more SH3 domains and proline-rich domains (PRDs), multivalent interactions of which are prerequisite for formation of LLPS alongside of intrinsically disordered regions.^12^^,^^13^^,^^14^ Indeed, recent studies have shown that Eps15 and FCHo, initiator proteins for clathrin-mediated endocytosis, undergo LLPS at the surface of the plasma membrane, implicating them in generation of membrane curvature and vesicle fission.^15^^,^^16^ Furthermore, some endocytic proteins listed above contain Bin/Amphiphysin/Rvs (BAR)-domains, which bind to lipid membranes *in vitro*,^17^ and also interact with synapsin.^18^^,^^19^^,^^20^ Thus, it is tempting to speculate that one of the endocytic proteins or a combination thereof have potential to undergo LLPS as synapsin does, and thereby contribute to their restricted localization in SV clusters, as well as formation of SV clusters in coordination with synapsin.

In this study, we demonstrate that endophilin A1 (EndoA1), known as an endocytic scaffold protein, forms highly condensed, self-organized assemblies via LLPS at physiologically plausible concentrations in presynaptic terminals. Although EndoA1 alone cannot promote SV-like vesicle clusters in COS7 cells, EndoA1 condensates drastically facilitate formation of synapsin droplets and exhibit potential to recruit various endocytic proteins, such as intersectin and dynamin. Furthermore, EndoA1 accumulates in SV-like clusters in a synapsin-dependent manner, suggesting that EndoA1 condensates are responsible for accumulation of other endocytic proteins in SV clusters. Consistent with liquid-like behaviors *in vitro* and in heterologous systems, EndoA1, positioned in SV clusters by LLPS, is dispersed from presynaptic terminals simultaneously with synapsin during repetitive stimulation, and is re-assembled after stimulation with kinetics similar to those of synapsin in cultured hippocampal neurons, indicating that cooperative behavior of EndoA1-and synapsin 1-condensates may serve an essential function in formation and reassembly of SV clusters in response to stimulation.

## Results

### Endophilin A1 undergoes LLPS *in vitro*

**Figure 1:**
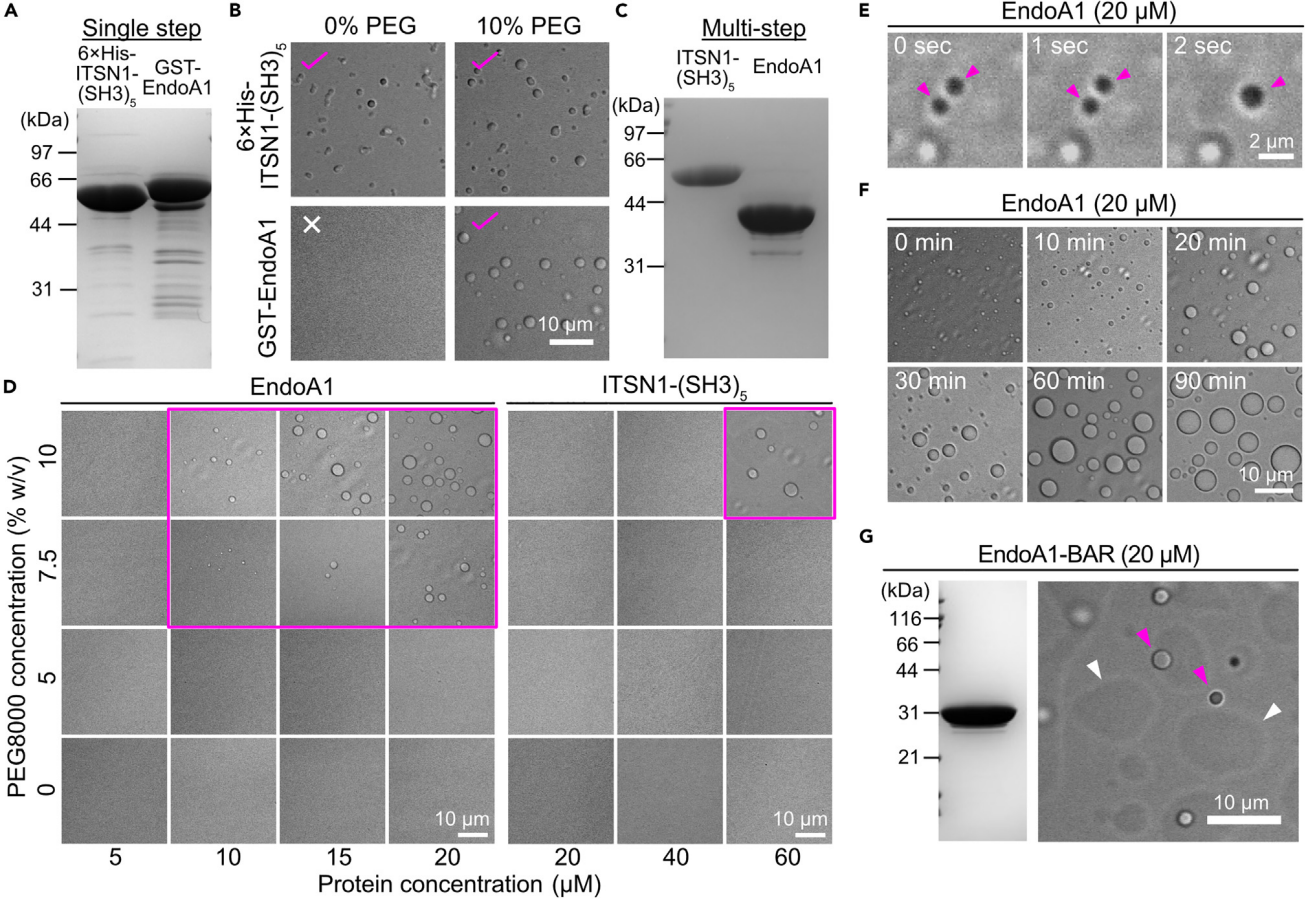
EndoA1, but not ITSN1-(SH3)_5_, can potentially undergo phase separation at physiologically relevant concentrations in presynaptic terminals (A) Coomassie blue staining of purified recombinant 6× His-intersectin 1 (ITSN1-(SH3)_5_) and GST-endophilin A1 (EndoA1) proteins with single-step affinity chromatography. 13.5 μg of recombinant proteins were loaded onto a 12.5% SDS-PAGE gel. Molecular weights are indicated on the left (kDa). (B) Phase separation assay for 6× His-ITSN1-(SH3)_5_ and GST-EndoA1 proteins. 6× His- ITSN1-(SH3)_5_ (20 μM) forms droplet-like structures irrespective of the presence of 10% PEG8000 (upper panels). On the other hand, GST-EndoA1 (20 μM) forms droplet-like structures only in the presence of PEG8000 (lower panels). All images were taken under differential interference contrast microscopy (DIC) after 60 min incubation at room temperature either in the absence (0%) or presence (10%) of PEG8000. (C) Coomassie blue staining of purified recombinant ITSN1-(SH3)_5_ and EndoA1 proteins after removal of affinity tags and multi-step column chromatography. 13.5 μg of recombinant proteins were loaded onto a 12.5% SDS-PAGE gel. (D) Phase diagrams of EndoA1 and ITSN1-(SH3)_5_ with various protein and PEG concentrations. Various concentrations of EndoA1 (5–20 μM) (left panels) and ITSN1-(SH3)_5_ (20–60 μM) (right panels) were mixed with varying concentrations of PEG8000 (0–10% (w/v)). Conditions in which droplets are clearly visible are indicated by magenta squares. (E) Time-lapse imaging shows the capacity of EndoA1 droplets (20 μM) to fuse, indicating liquid-like properties. Magenta arrowheads indicate two droplets that are merging. Images were taken under a DIC microscope after a few minutes of LLPS induction by adding PEG8000 (10% final). (F) Droplets of EndoA1 (20 μM) gradually grow after LLPS induction by addition of PEG8000 (10% final). Images were taken under a DIC microscope at each time point indicated at the upper left corner of each image. (G) Purification and droplet formation of EndoA1-BAR domain. (left panel) Coomassie blue staining of purified EndoA1-BAR domain (corresponding to amino acids 1–247). 13.5 μg of recombinant protein was loaded onto a 12.5% SDS-PAGE gel. (right panel) A representative image of the non-labeled EndoA1-BAR domain (20 μM). Magenta arrowheads indicate droplets floating in the solution, and white arrowheads indicate droplets settled onto the glass surface.

To examine whether soluble endocytic proteins have potential to undergo LLPS *in vitro*, we set up a series of expression vectors, either pGEX or pET, encoding FCHo2, FCHSD2, a full-length or a (SH3)_5_-domain of intersectin 1 (ITSN1-(SH3)_5_), endophilin A1 (EndoA1), amphiphysin, synaptojanin and dynamin 1, and expressed them in *E*. *coli*. These proteins potentially form a multivalent protein network,^21^ which represents the central characteristics of protein phase separation via distinctive domain structures (SH3 domain, proline-rich domain and μHD domain) (Figure S1).^13^^,^^21^^,^^22^ After single-step affinity column purification, we obtained GST-fused EndoA1 full-length and 6× His-tagged ITSN1-(SH3)_5_ with a high degree of purity and sufficient yields, whereas others were either poorly expressed, contained multiple degraded bands, or were partitioned into the insoluble fraction (Figure 1A). An initial test to examine their potential to form droplets under differential interference contrast (DIC) microscopy revealed that GST-EndoA1 formed micron-sized droplet-like structures in the presence of 10% polyethylene glycol (PEG), a widely used crowding reagent, at a final protein concentration of ∼20 μM, whereas 6× His-tag ITSN1-(SH3)_5_ (also at a final concentration of ∼20 μM) formed droplets irrespective of PEG (Figure 1B). To exclude undesired effects from affinity tags or minor contamination from host cells, we cleaved off affinity tags using proteases and removed contamination through further purification steps using an ion exchange column and/or a gel filtration column, resulting in much purer preparations (Figure 1C). Using these purified proteins, we then tested their potential to form droplet-like structures in various protein and PEG concentrations. Notably, in the presence of >7.5% PEG, EndoA1 formed clear droplets at protein concentrations above 10 μM, which was the physiologically relevant concentration in synaptosomes derived from rat cortex (16 μM)^23^ (Figure 1D, left). On the other hand, ITSN1-(SH3)_5_ lacking 6× His-tag did not form clear droplets at its physiological concentration (20 μM) even in the presence of 10% PEG, and formed clear droplets only at 60 μM, far beyond its concentration in synaptosomes^23^ (Figure 1D, right). These droplets spontaneously merged (Figure 1E for EndoA1), progressively increasing in size up to 10 μm during incubation up to ∼60 min at room temperature (Figure 1F).

**Figure 2:**
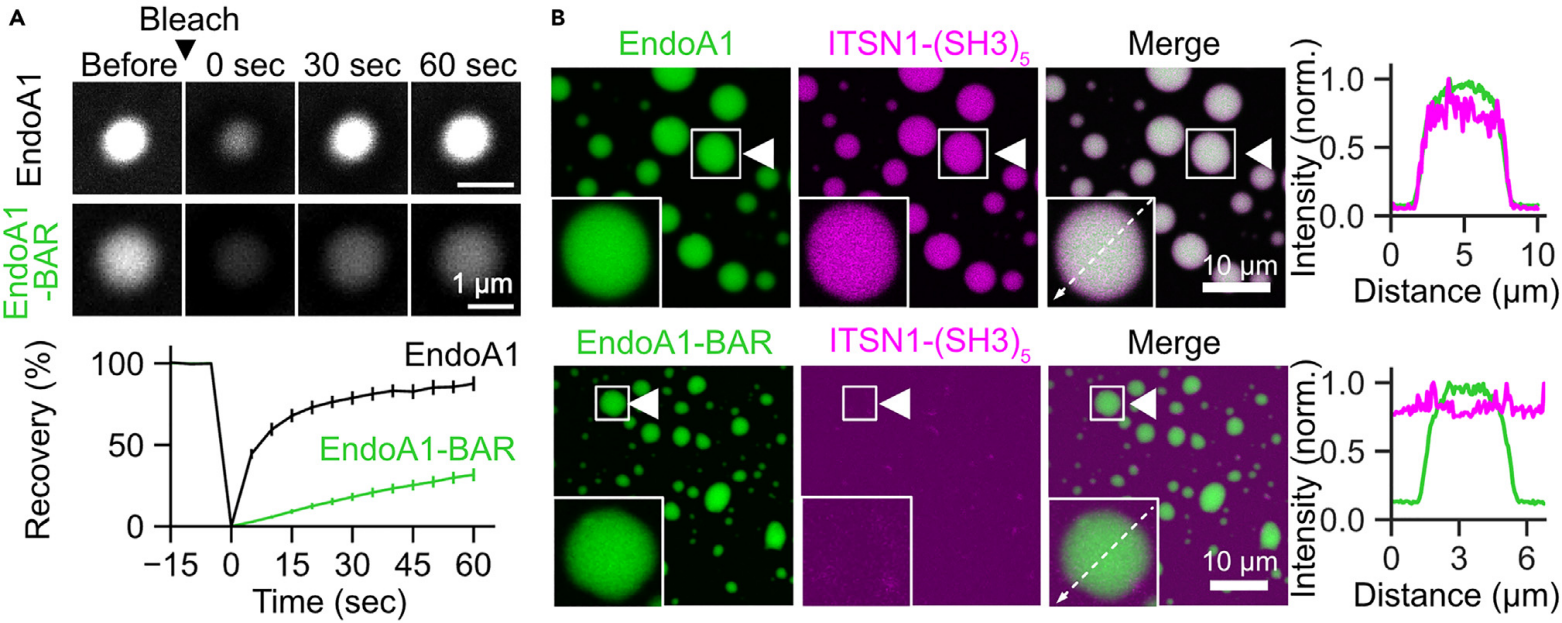
EndoA1 droplets exhibit FRAP and recruit ITSN1-(SH3)_5_ (A) Fluorescence recovery after photobleaching (FRAP) of EndoA1-full-length (black trace) and EndoA1-BAR (green trace) droplets. Images were taken at 0.2 Hz. Each data point and the respective error bar represent the means and standard errors of the means (s.e.m): n = 14 droplets (EndoA1) and n = 17 droplets (EndoA1-BAR). (B) EndoA1 droplets recruit ITSN1-(SH3)_5_ via its SH3-domain. (upper panel) EndoA1-full length (20 μM, green) was mixed with 20 μM ITSN1-(SH3)_5_ (magenta). The right line scan traces show perfect colocalization of full-length EndoA1 with ITSN1-(SH3)_5_. (lower panel) EndoA1-BAR droplets do not recruit ITSN1-(SH3)_5_. EndoA1-BAR (20 μM, green) was mixed with 20 μM ITSN1-(SH3)_5_ (magenta). The right line scan traces indicate no accumulation of ITSN1-(SH3)_5_ in EndoA1-BAR droplets.

To determine which part of EndoA1 accounts for its capacity to undergo LLPS, we prepared its N-BAR domain (aa 1–247) and tested whether it formed droplets (Figure 1G). At 20 μM in the presence of 10% PEG, we observed clear droplet-like structures, indicating that the BAR domain alone sufficed to form condensates. Notably, EndoA1-BAR protein formed much larger amorphous structures on the surface of a coverslip which were not seen in the case of full-length EndoA1, indicating a contribution of the C-terminus of EndoA1 to define biophysical properties of EndoA1 droplets (see also Figure 2).

To further validate the potential of EndoA1 to form droplets via LLPS, and its ability to recruit ITSN1, which reportedly interacts with EndoA1 through SH3-SH3 interactions,^24^ we covalently labeled EndoA1 and ITSN1-(SH3)_5_ proteins with Cy5 and Cy3 mono-reactive dye, respectively. Fluorescence recovery after photobleaching (FRAP) experiments using Cy5-labeled EndoA1 revealed near full recovery within 1 min, indicating that EndoA1 droplets are indeed formed by LLPS (Figure 2A). In contrast, FRAP of Cy5-labelled EndoA1-BAR domain exhibited much slower recovery than that for full-length EndoA1 (Figure 2A), indicating the rest of the EndoA1 protein, e.g., the SH3 domain, may help to determine the biophysical properties of EndoA1 condensates. When Cy5-labelled EndoA1 was mixed with Cy3-labelled ITSN1-(SH3)_5_ (both at 20 μM in the presence of 10% PEG, in which ITSN1-(SH3)_5_ by itself did not undergo phase separation, Figure S2), ITSN1-(SH3)_5_ formed spherical droplets that perfectly co-localized with EndoA1 (Figure 2B). In contrast, the BAR domain of EndoA1, lacking the SH3 domain, did not recruit ITSN1-(SH3)_5_ into the droplets (Figure 2B), suggesting that the SH3 domain of EndoA1 is responsible for recruiting ITSN1 into the condensed phase. It should be noted that the characteristics of EndoA1 and its BAR domain that enable it to undergo LLPS are fully compatible with recent observations by Mondal et al., in which formation of EndoA1 condensates with its binding partners via multivalent interactions are implicated in fast EndoA1-mediated, clathrin-independent endocytosis operating at plasma membranes of non-neuronal cell types.^25^ However, the endogenous expression level of EndoA1 in non-neuronal cells, which might be one of the key parameters to define the ability of proteins to phase separate in a physiological context, were not carefully taken into account in their study (see also discussion).

### EndoA1 forms liquid-like droplets in COS7 cells

**Figure 3:**
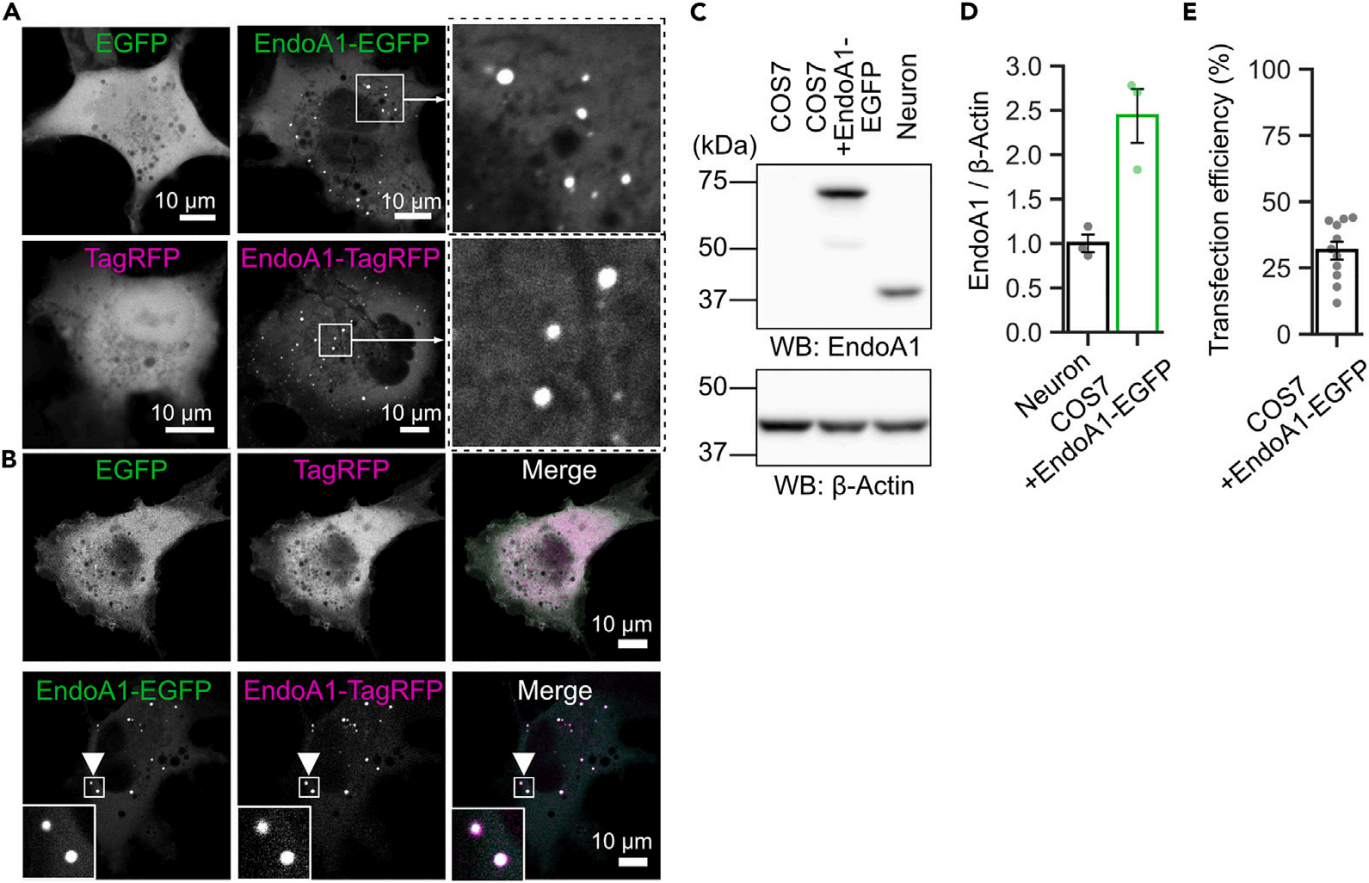
EndoA1 forms cytoplasmic condensates when expressed in COS7 cells (A) EndoA1 fused with fluorescent proteins (EGFP or TagRFP) forms cytoplasmic droplet-like structures in COS7 cells. Representative images of COS7 cells transfected either with EndoA1-EGFP or EndoA1-TagRFP are shown (middle). Cells transfected either with EGFP alone or TagRFP alone are shown in left panels as controls. Right-side images show magnified views of framed areas in the middle images. (B) Co-expression of EndoA1-EGFP and EndoA1-TagRFP results in droplet-like condensates that contain both proteins (bottom). On the other hand, co-expression of EGFP and TagRFP does not result in droplet-like structures (upper images). (C) Western blot analysis to estimate EndoA1-EGFP level in COS7 cells in comparison to non-transfected COS7 cells and lysates extracted from mouse cultured hippocampal neurons (14 days *in vitro*). 20 μg of lysates were subjected to western blotting with an anti-EndoA1 antibody (top). The band corresponding to EndoA1-EGFP appears at ∼70 kDa, whereas endogenous EndoA1 in neuronal lysates appears at ∼40 kDa. β-actin antibody was taken as a loading control, and band intensities were used for normalization (bottom). (D) Quantification of EndoA1-immunoreactive bands in each lane shown in (C). The bar graph represents the mean ± s.e.m from 3 independent samples. Values were normalized to those of lysates from neural cultures. The band intensity of EndoA1 in COS7 cells transfected with EndoA1-EGFP was 2.4 ± 0.3 times higher than that of neuronal cultures. (E) Estimation of transfection efficiency of COS7 cells with EndoA1-EGFP. To estimate expression levels of EndoA1-EGFP in individual COS7 cells more precisely, transfection efficiency in our heterologous expression system was determined. The number of COS7 cells transfected with the EndoA1-EGFP was determined by the EGFP signal, whereas total cell numbers were counted by staining cells with Hoechst-33342. The bar graph represents the mean ± s.e.m from 11 independent fields. Transfection efficiency was 31.5 ± 3.4%.

Consistent with results *in vitro* with purified proteins, EndoA1 C-terminally tagged either with EGFP or TagRFP formed droplet-like structures in cytoplasm when expressed in COS7 cells, whereas EGFP or TagRFP alone did not (Figure 3A). Co-expression of EndoA1 tagged with different fluorescent proteins resulted in condensates that contained both (Figure 3B), ruling out a potential artifact that weak homophilic interactions between the respective tags facilitated droplet formation. These droplets exhibited FRAP, albeit more slowly and to a lesser extent than those observed *in vitro* (Figure S3). Although inspection of EndoA1 levels in COS7 cells by immunoblotting suggested that EndoA1-EGFP expression was ∼8-fold higher than that observed in lysates extracted from cultured hippocampal neurons (Figures 3C–3E), the expression level of EndoA1-EGFP in COS7 cells might not be much above its endogenous EndoA1 levels in presynaptic terminals, because EndoA1 is substantially enriched in presynaptic terminals,^26^ (also see below). It should be emphasized here that endogenous EndoA1 in COS7 cells eluded detection, suggesting that the endogenous EndoA1 level is not sufficiently high to undergo LLPS in the cytoplasm of fibroblast cells.

### EndoA1 is recruited into SV-like vesicle clusters in a synapsin-dependent manner in COS7 cells

The ability of EndoA1 to form condensates in COS7 cells enabled us to examine possible contributions of EndoA1 in SV cluster formation, using those cells as a model. A previous study demonstrated that co-expression of synapsin and tag-free synaptophysin (Syph) induces formation of SV-like vesicle clusters in the cytoplasm, whereas Syph C-terminally tagged with EGFP alone also induced similar clusters, presumably owing to the property of EGFP to form weak dimers (Figure S4).^6^ Of interest, despite the ability of synapsin to undergo LLPS *in vitro*,^4^ expression of synapsin alone in COS7 cells does not show droplet-like appearance, but rather shows a coarse cytoplasmic distribution.^6^ Thus, this system is well suited for testing possible contributions of EndoA1 in modulation of synapsin-LLPS, as well as in formation of SV-like vesicle clusters, either dependent on synapsin, or not.

**Figure 4:**
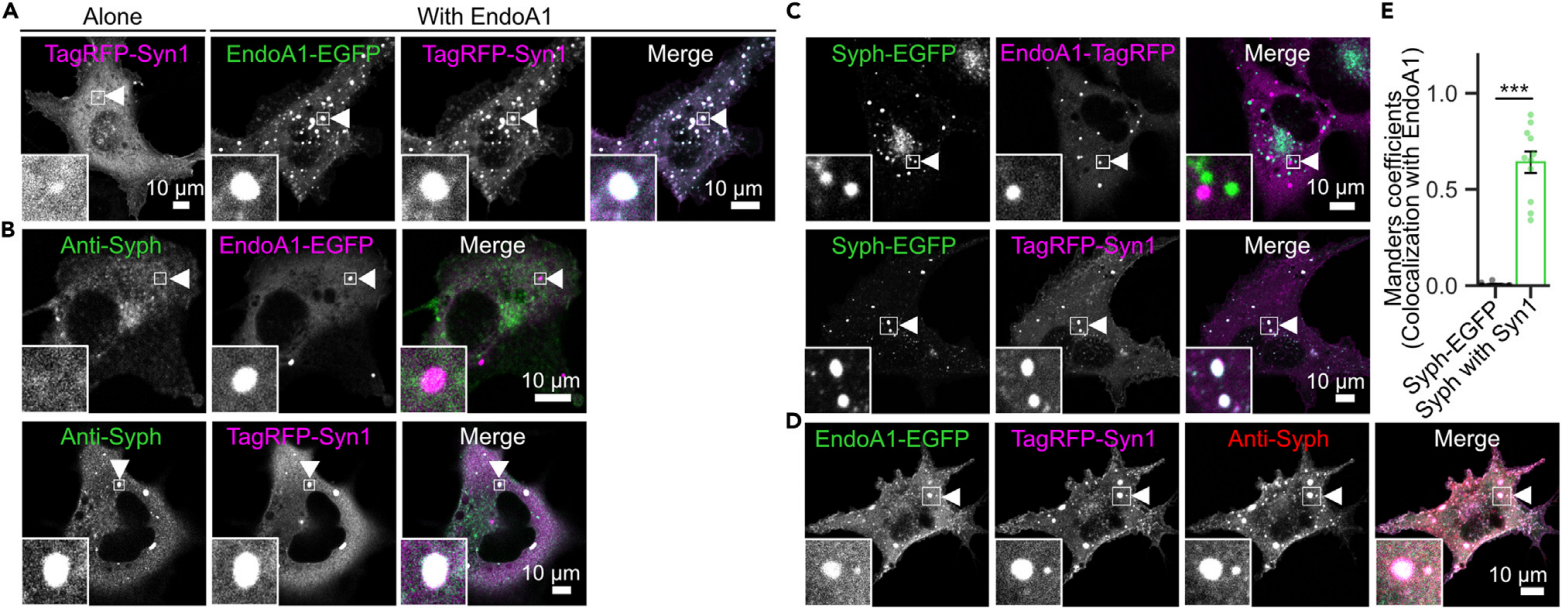
EndoA1 promotes formation of synapsin condensates and is recruited into SV-like vesicle clusters in a synapsin-dependent manner in COS7 cells (A) EndoA1 facilitates droplet formation of synapsin in COS7 cells. Expression of TagRFP-synapsin 1 alone shows a coarse cytoplasmic distribution in COS7 cells (left). However, co-expression of EndoA1-EGFP (green) with TagRFP-synapsin 1 (magenta) drastically promotes formation of cytoplasmic droplets that contain both proteins (right panels). (B) Synapsin, but not EndoA1, promotes clustering of SV-like vesicles in COS7 cells. (upper panels) EndoA1-EGFP (shown in magenta) forms droplets in COS7 cells, but does not promote SV-like clusters of Syph-positive puncta (Syph, shown in green). (lower panels) Co-expression of TagRFP-synapsin 1 (magenta) and tag-free synaptophysin (Syph, green) forms SV-like clusters structures in COS7 cells (see also Figure 5). Tag-free Syph was visualized by immunostaining after fixation. (C) Synapsin, but not EndoA1, is recruited into SV-like clusters induced by expression of Syph-EGFP in COS7 cells. (upper panels) EndoA1-TagRFP (magenta) forms independent droplets from Syph-EGFP puncta (green). (lower panels) TagRFP-synapsin 1 (magenta) colocalizes with Syph-EGFP puncta (green). (D) EndoA1-EGFP co-assembles with TagRFP-synapsin 1 and tag-free Syph in COS7 cells. After fixation, cells were immunostained with Syph antibody, and fluorescence of EndoA1 (green), synapsin 1 (magenta) and Syph (red) was imaged. (E) Quantification of EndoA1 droplets co-localized either with Syph-EGFP or with tag-free Syph co-expressed with synapsin 1 (Syn1). Colocalization analysis was performed using the Manders coefficient. Values are mean ± s.e.m. with n = 9 images and n = 11 images for Syph-EGFP/EndoA1-TagRFP and tag-free Syph/TagRFP-Syn1/EndoA1-EGFP, respectively. ∗∗∗p < 0.001, unpaired *t*-test.

First, based on biochemical data showing a direct interaction between EndoA1 and synapsin,^19^ we examined whether co-expression of EndoA1 had any impact on synapsin in COS7 cells (Figure 4A). Consistent with a previous study,^6^ TagRFP-tagged synapsin 1 exhibited a coarse cytoplasmic distribution without clear signs of droplet-like puncta. However, co-expression of EndoA1-EGFP drastically promotes synapsin droplets in the cytoplasm that perfectly colocalize with EndoA1 (Figure 4A).

Next, we examined whether EndoA1 can promote accumulation of SV-like vesicles into clusters. To this end, either EndoA1-EGFP or TagRFP-synapsin 1 was co-expressed with tag-free Syph (Figure 4B). Although synapsin facilitated formation of SV clusters marked by Syph antibody as previously reported,^6^ EndoA1 condensates did not accumulate Syph-carrying vesicles into the droplets (Figure 4B). Moreover, when EndoA1 was co-expressed with Syph-EGFP, EndoA1 formed droplet-like structures independent of Syph-EGFP positive vesicle clusters (Figures 4C and 4E), whereas synapsin was co-localized with Syph-EGFP-positive puncta as previously reported.^6^ Finally, when EndoA1 was co-expressed with synapsin and tag-free Syph, EndoA1 was effectively recruited into synapsin/Syph double-positive puncta (Figures 4D and 4E). These results demonstrate that although EndoA1 alone does not suffice to form SV clusters, it is recruited into SV clusters in a synapsin-dependent manner and can cause synapsin to undergo LLPS.

### EndoA1 and synapsin form cytoplasmic condensates independent of SV-like vesicle clusters

**Figure 5:**
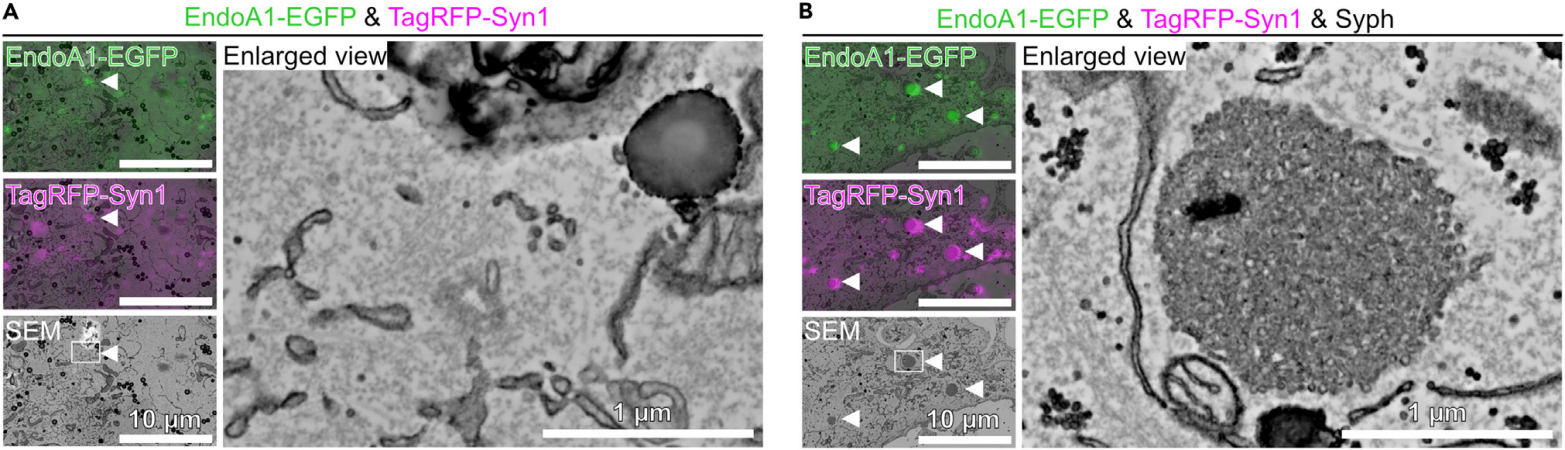
Correlative light and electron microscopy reveals membrane-free condensates that contain EndoA1 and synapsin (A) Representative fluorescence images for EndoA1-EGFP and TagRFP-synapsin 1 (Syn1) and a scanning electron microscopic (SEM) image of the corresponding region (left panels). Putative droplets containing both proteins are indicated by arrowheads. The right panel shows a magnified view of an area indicated by a white square. Note that no visible membrane structures are seen in putative condensates consisting of EndoA1 and synapsin. (B) Representative fluorescence images for EndoA1-EGFP and TagRFP-Syn1 in which tag-free Syph was also transfected (left). The SEM image of the corresponding area is shown at the bottom, where putative droplets containing EndoA1, Syn1 and also Syph (see Figure 4D) are indicated by arrowheads. The right panel shows a magnified view of the area indicated by a white square. Note that EndoA1/Syn1-double-positive fluorescent puncta contain electron dense structures, which likely represent clusters of small vesicles with a diameter of ∼40 nm.

BAR-domain containing proteins, including EndoA1, bind and tubulate lipid membranes *in vitro* and in heterologous cells.^27^^,^^28^^,^^29^ When present at high concentrations, e.g., 40 μM, however, the BAR domains can produce small vesicles, e.g., ∼50 nm in diameter, from larger liposomes.^30^ Moreover, endophilin has been implicated in one of the clathrin-independent endocytosis modes, i.e., the Fast Endophilin Mediated Endocytosis (FEME).^31^ Given the potential of synapsin to recruit negatively charged vesicles^4^ and to coalesce with EndoA1 (Figure 4A), it is therefore possible that overexpression of EndoA1-EGFP in COS7 cells could facilitate the intrinsic FEME, resulting in hyper-production of small vesicles that could then be recruited into cytoplasmic EndoA1 condensates, in particular, with the aid of synapsin. To examine this possibility, a correlative light and electron microscopy (CLEM) analysis was performed to visualize membrane structures within the droplets that were fluorescently labeled with proteins of interest. For comparisons, two conditions were examined, i.e., co-expression of EndoA1 and synapsin 1 either in the absence or presence of tag-free Syph, corresponding to the conditions shown in Figures 4A and 4D, respectively. When EndoA1 and synapsin were co-expressed, we did not detect any membranous structures that correlated well with positions of fluorescence-positive puncta throughout Z-stacks (Figure 5A and VideoS1). In stark contrast, when tag-free Syph was transfected in addition, the fluorescent puncta that were positive both for synapsin and EndoA1 contained highly electron-dense structures, which appeared under higher magnification to be clusters of small SV-like vesicles, consistent with a previous observation^6^ (Figure 5B and VideoS2). These results not only confirm that EndoA1 indeed co-assembles with SV-like vesicle clusters induced by synapsin and Syph, but also indicate that EndoA1 can form condensates that are devoid of small vesicle membranes even in the presence of synapsin in a cellular context.

Video S1. A representative cell expressing EndoA1-EGFP and TagRFP-synapsin 1 imaged with correlative light and electron microscopy This video shows serial SEM images overlaid with EndoA1-EGFP fluorescence. Two regions where EndoA1 droplets are located are enlarged related to Figure 5.

Video S2. A representative cell expressing EndoA1-EGFP, TagRFP-synapsin 1 and tag-free Syph imaged with correlative light and electron microscopy This video shows the serial SEM images overlaid with EndoA1-EGFP fluorescence. A region where EndoA1 droplet is located is enlarged. Note that the fluorescence-positive droplet-like structure contains an electron-dense vesicle cluster related to Figure 5.

### EndoA1 condensates recruit ITSN1, dynamin, and amphiphysin that are not accumulated into SV-like vesicle clusters by themselves

**Figure 6:**
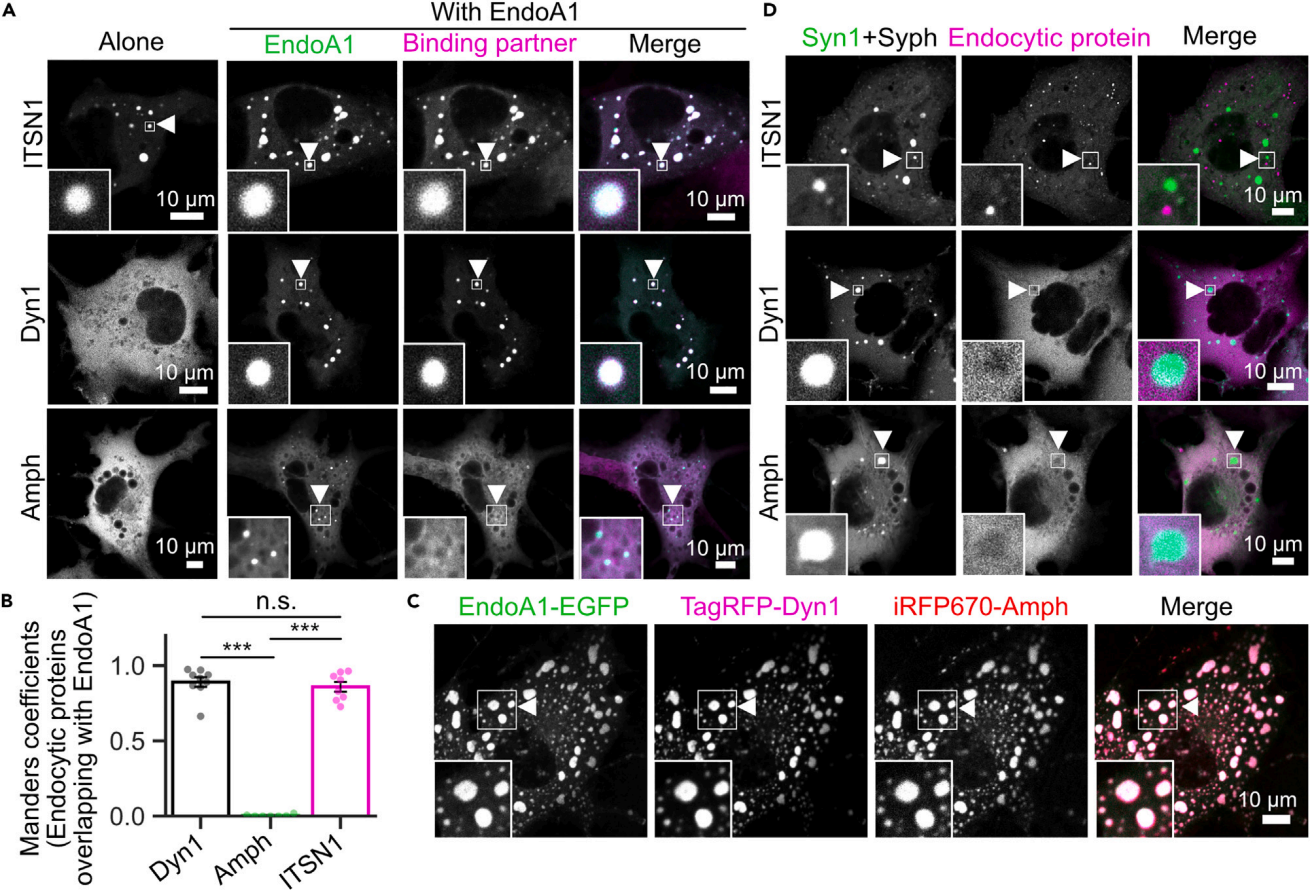
EndoA1 condensates potentially recruit various endocytic proteins that by themselves are not accumulated into SV-like vesicle clusters (A) EndoA1 droplets recruit dynamin and ITSN1, but not amphiphysin in COS7 cells. COS7 cells were either transfected with individual endocytic proteins (ITSN1, dynamin 1, or amphiphysin (Amph) or co-transfected with EndoA1-EGFP. Insets are magnified views of areas indicated by arrowheads. (B) Quantification of colocalization of EndoA1 with endocytic proteins shown in (A). Values indicate means ± s.e.m. with n = 9 images, n = 7 images, and n = 8 images for Dynamin, Amph, and ITSN1, respectively. ∗∗∗p < 0.001, ns, not significant, one-way ANOVA followed by the Tukey–Kramer post-hoc test. (C) Amph co-assembles with EndoA1 droplets through dynamin 1. Triple-transfection of EndoA1 (green), dynamin 1 (magenta), and Amph (red) in COS7 cells results in complete colocalization of the three proteins. (D) ITSN1, dynamin and Amph are not recruited into SV-like vesicle clusters induced by Syn1 + Syph. COS7 cells were triple-transfected with each endocytic protein plus synapsin + tag-free Syph (Syn1 + Syph). Insets are magnified views of areas indicated by arrowheads. Note that, unlike EndoA1, none of these proteins co-assemble with Syn1 + Syph.

As indicated by *in vitro* experiments with EndoA1 and ITSN1-(SH3)_5_ (Figure 2) as well as the ability of EndoA1 to recruit synapsin into condensates in COS7 cells (Figure 4A), it is plausible that EndoA1 condensates also recruit its binding partners, including some endocytic proteins of which we failed to obtain pure protein preparations with the *E*. *coli* expression system. When dynamin 1 or amphiphysin (Amph) tagged with a fluorescent protein was transfected in COS7 cells, both proteins showed dispersed cytoplasmic distributions without visible droplet-like structures (Figure 6A). In contrast, EGFP-ITSN1, a full-length long-splicing variant, formed spherical droplets in the cytoplasm (Figure 6A), likely reflecting the potential of ITSN1-(SH3)_5_ to undergo LLPS at high concentrations (Figure 1D). When co-expressed with EndoA1, both dynamin 1 and ITSN1 formed droplets together with EndoA1, whereas Amph did not (Figures 6A and 6B). The latter was unexpected, but an additional transfection of dynamin robustly promoted formation of droplet-like structures that contained Amph, dynamin and EndoA1 (Figure 6C). These results demonstrate that EndoA1 condensates serve as a reservoir for other endocytic proteins through direct or indirect interactions that could form multivalent molecular networks among endocytic proteins.^22^

As suggested by the above observations, we then examined whether ITSN1, dynamin, or Amph can be recruited into SV-like vesicle clusters like EndoA1. Despite the potential of ITSN1 and Amph to interact with synapsin,^18^^,^^20^ however, none of them were recruited into SV-like vesicle clusters induced by synapsin 1 and tag-free Syph (Figure 6D). Notably, despite *in vitro* observations that a recombinant ITSN1-(SH3)_5_ fragment was recruited into synapsin droplets,^4^ full-length ITSN1 formed cytoplasmic droplets that were independent of SV-like clusters marked by synapsin/Syph. Thus, these results collectively indicate that localization of these endocytic proteins in SV clusters depends critically on the presence of EndoA1.

### EndoA1 localizes within SV clusters at hippocampal synapses

**Figure 7:**
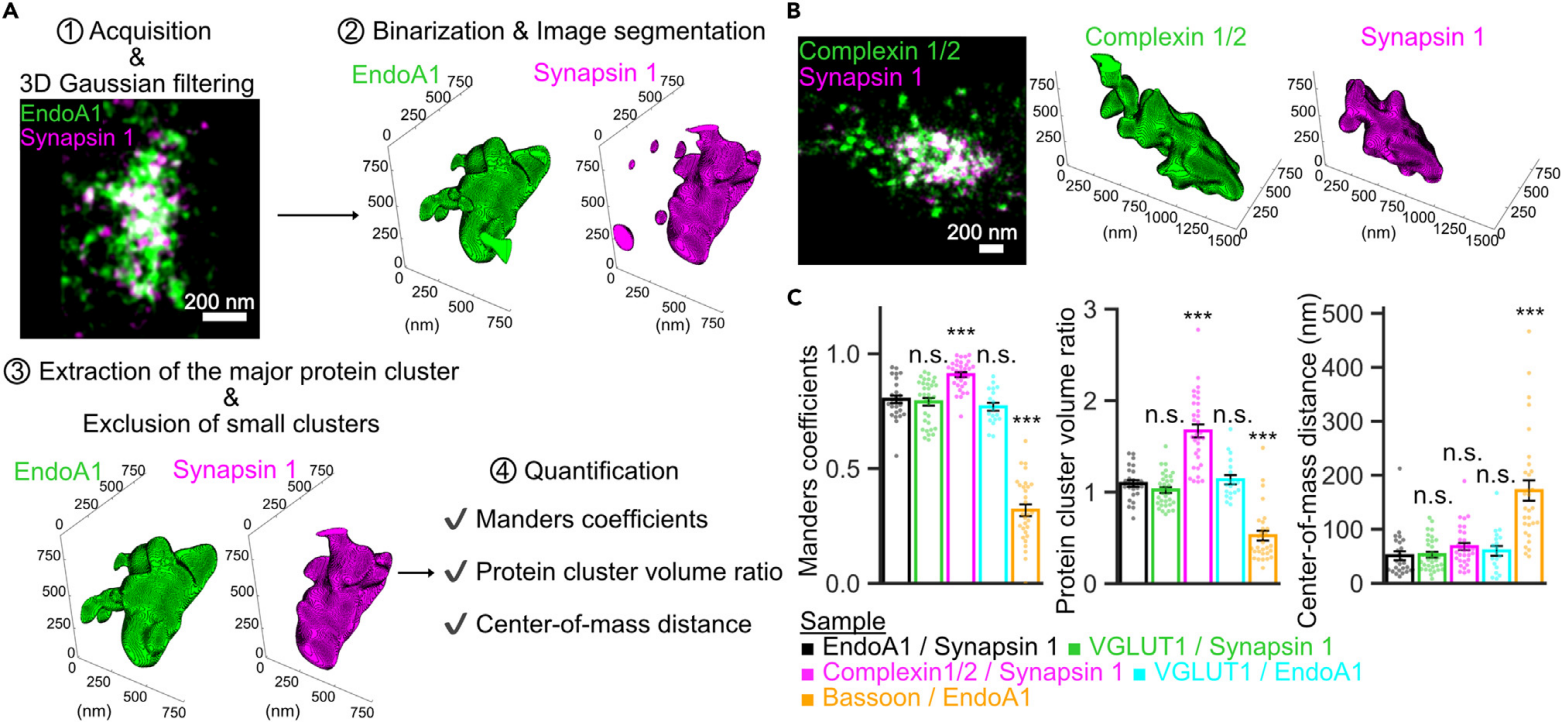
3D-STORM analysis reveals restricted distribution of EndoA1 in SV clusters at cultured hippocampal neurons (A) The workflow for colocalization analysis of two synaptic proteins by 3D-STORM analysis. The top left panel shows a representative 2D projected two-color STORM image of EndoA1 (green) and synapsin 1 (magenta) in a single synaptic bouton. (1) 3D-STORM images were processed with a 3D Gaussian filter. (2) After binarization, positive voxels were then divided into connected components and labeled as clusters. (3) One major cluster for each synaptic protein was extracted for the following analysis while all other small clusters were excluded. (4) The center-of-mass and the volume of each protein cluster were determined by calculating mean voxel coordinates and total voxels of extracted major clusters for each synaptic protein, respectively. Manders coefficients were quantified by calculating the ratio of overlapping voxels to total voxels of the reference protein cluster. (B) (left panels) A representative 2D projected two-color STORM image of complexin 1/2 (green) and synapsin 1 (magenta) in a single synaptic bouton. Complexin 1/2 immunoreactivity is distributed more widely than synapsin 1 signals and wraps around the synapsin 1 cluster. (right panels) 3D visualization of the complexin 1/2 (green) and synapsin 1 (magenta) major clusters in a single synaptic bouton. (C) Quantification of Manders coefficients, volume ratios, and the center-of-mass distance between two synaptic protein clusters. Synaptic molecular combinations quantified were described on left panels. Bar graphs represent means ± s.e.m. for EndoA1/synapsin 1 (black, n = 26 synapses), VGLUT1/synapsin 1 (green, n = 33 synapses), complexin1/2 / synapsin 1 (magenta, n = 32 synapses), VGLUT1/EndoA1 (cyan, n = 19 synapses) and bassoon/EndoA1 (orange, n = 30 synapses). ∗∗∗p < 0.001, ns, not significant. one-way ANOVA followed by the Tukey–Kramer post-hoc test.

The above results *in vitro* and in COS7 cells support the notion that multiple endocytic proteins are clustered in SV clusters, aided by phase separation of synapsin and EndoA1 through multivalent interactions. To gain further insights into EndoA1 condensates in mammalian central synapses, which exhibit more complex membrane organization than lamprey synapses, we attempted to map EndoA1 proteins related to SV clusters in fixed cultured hippocampal neurons in greater detail by employing a three-dimensional stochastic optical reconstruction microscope (3D-STORM)^32^ combined with a quantitative framework to evaluate colocalization of two synaptic proteins in the same preparation (Figure 7A). To validate EndoA1 localization in presynaptic terminals, synapsin 1, VGLUT1, complexin and bassoon were chosen as specific markers for SV clusters, a presynaptic soluble protein and active zones.^4^^,^^33^^,^^34^^,^^35^ We compared spatial relationships of 5 pairs of synaptic proteins listed in Figure 7C by evaluating three parameters; i.e., Manders coefficients,^36^ volume ratios, and the center-of-mass distance between the two synaptic protein clusters (Figure 7C). Such analyses revealed that (1) protein clusters of EndoA1, synapsin1, and VGLUT1 were highly co-localized (>80%), (2) volumes occupied by EndoA1, VGLUT1, and synapsin 1 were identical, as evidenced by respective cluster volume ratios close to 1.0 (1.15 ± 0.04 for EndoA1/synapsin 1, n = 25 synapses; 1.02 ± 0.03 for VGLUT1/synapsin 1, n = 33 synapses; 1.13 ± 0.05 for VGLUT1/EndoA1, n = 19 synapses) and (3) center-of-mass distances were not statistically different (Figures 7C and S5), indicating that spatial distributions of the three proteins are almost identical. In stark contrast, comparisons of complexin 1/2 and synapsin 1 revealed that the volume ratio of complexin 1/2 clusters to synapsin 1 clusters was significantly higher than 1 (1.68 ± 0.08 for complexin1/2 / synapsin1, n = 32 synapses). Considering the short center-of-mass distance (∼50 nm) and the high colocalization ratio (∼90%) between them, it is conceivable that synapsin 1, and therefore also EndoA1 and VGLUT1, partially occupy complexin 1/2 clusters. Given the volume ratio between complexin 1/2 to synapsin (1.68), and assuming that complexin 1/2 is evenly distributed in presynaptic terminals, SV clusters containing synapsin, as well as VGLUT1 and EndoA1, would occupy ∼60% of total volume of presynaptic terminals. As expected, quantification of bassoon clusters relative to EndoA1 with STORM showed much less overlapping distribution of these proteins with ∼200 nm of center-of-mass distance and only ∼30% cluster overlap (Figures 7C and S5).

### EndoA1 exhibits liquid-like properties in cultured hippocampal neurons

**Figure 8:**
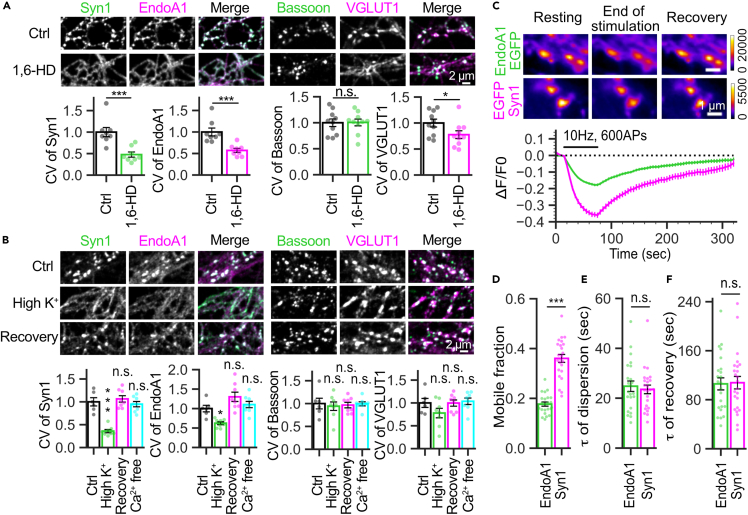
Activity-dependent dispersion and condensation cycles of EndoA1 and synapsin condensates in hippocampal neurons (A) EndoA1 exhibits liquid-like properties in cultured mouse hippocampal neurons. After 1 min exposure with 3% (w/v) 1,6-hexanediol (HD), neurons were immediately fixed and subjected to immunostaining. Images show immunolabeling of synapsin 1 (green), EndoA1 (magenta), bassoon (green) and VGLUT1 (magenta) in hippocampal neurons without 1,6-HD treatment (Ctrl, upper panels) and after 1min 1,6-HD treatment (1,6-HD, lower panels). To evaluate the extent of dispersion from presynaptic terminals, coefficients of variation (CVs) of manually defined lines along axon-like structures were determined (see STAR Methods for details), and were normalized to a control without 1,6-HD treatment). Values are normalized means ± s.e.m with n = 8 images and n = 10 images for synapsin 1-EndoA1 staining and Bassoon-VGLUT1 staining, respectively. ∗p < 0.05, ∗∗∗p < 0.001, ns, not significant, unpaired *t*-test. (B) EndoA1 and synapsin 1 dispersed from presynaptic terminals upon high K^+^ stimulation, and subsequently recovered after 10 min. Images show representative immunolabeling of synapsin 1 (green), EndoA1 (magenta), bassoon (green) and VGLUT1 (magenta) without stimulation (Ctrl, top), fixed immediately after 1 min high K^+^ (45 mM) treatment (High K^+^, middle) and fixed at 10 min after treatment, when high K^+^ solution was changed to ACSF (Recovery, bottom). Values are means ± s.e.m. for synapsin 1 and EndoA1 (n_Ctrl_ = 6 images, n_High K_^+^ = 8 images, n_Recovery_ = 8 images, n_Ca_^2+^_free_ = 8 images), for bassoon and VGLUT1 (n_Ctrl_ = 6 images, n_High K_^+^ = 8 images, n_Recovery_ = 8 images, n_Ca_^2+^_free_ = 8 images) normalized to the control. ∗p < 0.05, ∗∗∗p < 0.001, ns, not significant. one-way ANOVA followed by the Tukey–Kramer post-hoc test. (C) Live fluorescence imaging of EndoA1-EGFP or EGFP-Syn1 in cultured hippocampal neurons. These pseudo-color images show punctate signals of EndoA1-EGFP or of EGFP-Syn1 dispersed upon intensive electrical stimulation (10Hz, 600APs). Representative images at the start point (Resting), the endpoint of stimulation (End of stimulation) and the endpoint of imaging (Recovery). The horizontal pseudo-color scale shows fluorescence intensity values of EGFP fused with the respective proteins (arbitrary units). Bottom traces show the time course of EndoA1-EGFP (green) or EGFP-Syn1 (magenta) fluorescence changes in punctate structures during and after an electrical stimulation (10 Hz, 600APs). Images were taken at 0.2 Hz using an epifluorescence microscope. (D) Quantification of EndoA1-EGFP and EGFP-Syn1 mobile fractions. The bar graph shows that the mobile fraction of EGFP-Syn1 (0.36 ± 0.02) is larger than that of EndoA1-EGFP (0.18 ± 0.01). ∗∗∗p < 0.001, unpaired *t*-test. (E) Quantification of the time constant (τ) of EndoA1-EGFP and EGFP-Syn1 dispersion. The bar graph shows that the time constant of EndoA1-EGFP (24.8 ± 2.1 s) and EGFP-Syn1 (23.5 ± 1.7 s) dispersion are identical. ns, not significant, unpaired *t*-test. (F) Quantification of the time constant (τ) of EndoA1-EGFP and EGFP-Syn1 recovery. The bar graph shows that the time constant of EndoA1-EGFP (105.9 ± 9.9 s) and EGFP-Syn1 (107.8 ± 10.2 s) recovery are identical. ns, not significant, unpaired *t*-test. (C–F) The data shown represent the mean ± standard errors of the means (s.e.m) with n = 23 images and n = 24 images for EndoA1-EGFP and EGFP-Syn1, respectively.

Evidence for EndoA1 and synapsin to form condensates in living synapses is still lacking. To examine this, cultured hippocampal neurons were exposed to a solution containing 3% 1,6-hexanediol (1,6-HD), which has often been used to disrupt biomolecular condensates,^12^ for 1 min and were immediately fixed for immunolabeling. After this treatment, the punctate appearance of EndoA1, as well as that of synapsin, disappeared, as evident from significant decreases in the coefficient of variation of the fluorescent signals (Figures 8A and S6). Immunolabeling of VGLUT1, an SV marker in glutamatergic synapses was also dispersed, albeit to lesser extent, supporting the role of synapsin 1 to maintain SV clusters. On the other hand, immunolabeling of bassoon (an AZ marker) remained unaffected. Although it has been proposed that the AZ proteins, presumably including bassoon, are assembled with liquid-like condensates composed of various AZ proteins such as RIM and RIM-BP2,^35^^,^^37^ the resistance to 1,6-HD exposure in our conditions suggests that the molecular assembly of AZ proteins seems to be more rigid than that of SV clusters composed of SVs, synapsin and EndoA1.

As an alternative approach, we expressed EndoA1-EGFP or EGFP-synapsin 1 in cultured neurons, both of which showed punctate presynaptic localization, and monitored their behaviors in response to 3% 1,6-HD treatment for 1 min and thereafter. We confirmed that both proteins were dispersed rapidly upon addition of 1,6-HD, and recovered to their initial states within 5 min (Figure S6), indicating that both proteins indeed form condensates through LLPS in living synapses.

### Activity-dependent dispersion and reassembly of EndoA1 and its synchronicity with synapsin in cultured hippocampal neurons

Finally, we asked whether EndoA1 condensates would dynamically transform during neural activities, in which robust SV exocytosis, subsequent endocytosis, and SV reformation would be occurring continuously. It has been demonstrated that synapsin condensates formed *in vitro* as well as cytoplasmic droplets consisting of synapsin and Syph-laden vesicles in COS7 cells, dissociate on activation of CaMKII that phosphorylates synapsin, suggesting that activity-dependent Ca^2+^ influx into presynaptic terminals may trigger dissociation of synapsin condensates, and thereby, SV clusters.^4^^,^^6^ Indeed, synapsin was previously shown to exhibit activity-dependent dispersion from presynaptic terminals in cultured living neurons.^38^ To test whether EndoA1 also undergoes activity-dependent dispersion, we first exposed cultured neurons on coverslips to a high potassium solution (high K^+^) for 1 min, fixed them immediately or after 10 min, and subjected them to immunolabeling. Comparisons of the coefficient of variation of immunolabeling for synapsin and EndoA1 clearly indicate their activity-dependent dispersion from boutons and their subsequent recovery, whereas those of bassoon and VGLUT1 stayed within boutons (Figure 8B). Dispersion of synapsin and EndoA1 is clearly activity-dependent, because it was not observed in the absence of extracellular Ca^2+^. To examine further whether the activity-dependent dispersion and the subsequent recovery of EndoA1 are kinetically synchronized with those of synapsin, we performed live imaging of EndoA1-EGFP or EGFP-synapsin 1 expressed in cultured neurons (Figure 8B). Although the extent of dispersion from puncta was significantly larger for synapsin 1 than for EndoA1 during 600 action potentials (APs) at 10 Hz (Figures 8C and 8D), kinetics of dispersion during repetitive stimulation and of recovery after stimulation were indistinguishable (Figures 8C, 8E, and 8F).

## Discussion

Recent evidence has emerged to support a novel concept that various sub-compartments in presynaptic boutons are organized by phase separation of distinct protein components, such as synapsin for SV clusters and the major AZ proteins, such as RIM/RIM-BP2, liprin, and ELKS for AZ assembly.^39^^,^^40^^,^^41^ Our current results provide another example of biomolecular condensates in presynaptic terminals that enable condensation of various endocytic proteins within SV clusters. Our findings not only provide strong support for the series of electron microscopic observations in lamprey reticulospinal synapses that various endocytic proteins including endophilin reside in the SV clusters,^9^^,^^42^ but are also compatible with a proposal by Denker et al. that the resting pool of SVs functions as a ‘buffer’ for various soluble proteins associated with SV recycling.^43^ More importantly, our results indicate that there is a certain degree of hierarchy among endocytic proteins in terms of their properties to accumulate in SV clusters. Among proteins tested in this study, EndoA1 is the only protein that has potential to undergo phase separation at its physiologically relevant concentration at presynaptic terminals, to recruit various endocytic proteins into the condensates, and to accumulate in SV-like clusters via synapsin. In contrast, other endocytic proteins examined (dynamin 1, ITSN1 and amphiphysin) do not form condensates by themselves, but can be recruited into SV clusters aided by synapsin-EndoA1 condensates. Intriguingly, EndoA1 drastically drives synapsin to form condensates in COS7 cells, implicating that EndoA1 can modulate SV cluster formation through synapsin. These features of EndoA1 suggest that, in addition to its essential roles during SV endocytosis and reformation reported previously,^10^^,^^11^^,^^26^^,^^27^^,^^44^^,^^45^ EndoA1 serves a structural function in maintenance of dynamic SV clusters that commingle various endocytic proteins.

Although a recent study demonstrated the potential for EndoA1 to form condensates, which is fully compatible with our *in vitro* results,^25^ their functional implications differ substantially. First, although EndoA1 condensates are implicated in endophilin-mediated endocytosis in fibroblast cells, which takes place locally at the inner surface of plasma membrane in the size range of tens of nanometers,^25^ our results suggest that micron-sized EndoA1 condensates assemble various endocytic proteins in SV clusters, which fits well with the size of SV clusters in various types of synapses.^3^ Second, whereas our results revealed that EndoA1 by itself forms condensates at physiologically relevant cytoplasmic concentrations in presynaptic terminals (∼20 μM), much lower expression of all endophilin isoforms in fibroblast cells, e.g., <1 μM in total in HeLa cells^46^ (Figure 3) strongly suggest that it cannot form cytoplasmic condensates, at least by itself in fibroblast cells. Furthermore, synapsin that underlies formation of SV clusters is not expressed in fibroblast cells.^46^ Thus, the molecular assembly of synapsin and EndoA1 mediated by LLPS and described in this study may represent a structural specification of presynaptic terminals that is required for their peculiar functioning.

Because protein phase separation is highly dependent on concentrations of components, we speculate, according to our results with ITSN1, that EndoA1 may not be the only endocytic protein that functions as a ‘scaffold’ to recruit ‘client’ proteins into the condensates.^47^ Our results *in vitro* as well as in COS7 cells strongly suggest that ITSN1 can behave as another ‘scaffold’ protein especially when it is once recruited and condensed into EndoA1 droplets, in which the concentration of ITSN1 becomes much higher than when it is in diluted phase and may therefore exceed a critical concentration for ITSN1 to undergo LLPS. Likewise, other endocytic proteins not tested in this study also have potential to interact with EndoA1 and ITSN1 through multivalent interactions among SH3 domains, PRD and μHD domains.^21^ It is, therefore, conceivable that many, if not all, endocytic proteins at presynaptic terminals accumulate in SV clusters through phase separation of synapsin and various endocytic proteins, making a dynamic sub-cellular compartment that involves SV membranes and various protein condensates within presynaptic boutons.

Having said that, the peculiar ability of EndoA1 to facilitate formation of synapsin condensates in COS7 cells suggests that EndoA1 has the potential to modulate SV cluster formation and maintenance, primarily regulated by synapsin condensates. At lamprey synapses, however, injection of an antibody that recognizes the IDR of synapsin causes dispersal of distal SV clusters, whereas individual inhibition of interactions between synapsin and the SH3 domain of ITSN, amphiphysin, endophilin and syndapin does not cause vesicle dispersion at resting state,^7^^,^^44^^,^^48^ arguing against an essential role of those SH3 containing proteins in maintaining SV clusters under resting conditions. Nevertheless, because the synapsin IDR, self-interaction of which would be perturbed by the antibody, contains two PRDs that are responsible for the interactions with various SH3-containing proteins including EndoA1, a possibility cannot be completely ruled out that the antibody binding to the synapsin IDR would have interfered possible interactions with other proteins (e.g., endophilin) that would consequently affect liquid phase organization of SV clusters. With such technical difficulties to specifically interfere with individual protein-protein interactions in mind, it should be noted that injection of antibody that binds α-synuclein, which indeed promotes synapsin droplets on co-expression in COS7 cells as EndoA1 does, causes a severe depletion of SV clusters in lamprey synapses under resting conditions,^49^^,^^50^ indicating that other protein factors may be involved in formation and maintenance of SV clusters, presumably in a cooperative manner with synapsin.

Although it is difficult to test experimentally at this point, we envision the significance of EndoA1 condensates in synaptic physiology. First, by maintaining endocytic proteins at high concentrations in SV clusters near release sites, condensates might enable a rapid supply of those endocytic proteins to peri-active zones (endocytic hot spots) on demand. Consistent with this, EndoA1 condensates are dispersed, in parallel with synapsin condensates in response to stimulation (Figure 8), leading to an increase in concentrations of their constituents at peri-active zones. Subsequent reassembly of EndoA1 condensates would then lead to a reduction in cytoplasmic concentrations of EndoA1 and its ‘client’ endocytic proteins as endocytosis is being terminated. Such activity-dependent dispersion and reassembly of EndoA1 condensates involving other endocytic proteins may therefore constitute an elastic supramolecular assembly that enables activation of endocytic proteins in a manner that is coupled spatio-temporally with SV exocytosis.^51^ Furthermore, the ability of synapsin and EndoA1 to form membrane-free condensates in COS7 cells (Figure 5) suggests that these condensates, if they can form condensates outside of SV clusters, may serve as a molecular glue to catch newly regenerated SVs and transfer them back to existing SV clusters. The clathrin uncoating defects reported in endophilin triple KO mice,^11^ and the requirement of Syph in SVs to form vesicle clusters with synapsin^6^ collectively indicate that exposure of Syph on newly formed vesicles to the cytoplasm by shedding clathrin-coats may trigger the capture and subsequent recruitment of reformed SVs into SV clusters by synapsin-EndoA1 condensates. Fine manipulation of EndoA1 and synapsin condensates in living neurons, i.e., optogenetic tools for manipulating condensates, will certainly help to decipher the role of these condensates in SV recycling.

In summary, we propose that liquid-like protein assembly mediated by EndoA1 functions as a dynamic reservoir for multiple endocytic proteins to direct them into SV clusters formed by synapsin. During neural activity, EndoA1 is dispersed concomitantly with synapsin from SV clusters, and re-assembled as endocytosis is completed. Although it has long been proposed that such migration cycles of endocytic proteins between SV clusters and peri-AZs during synaptic activity may contribute to tight coupling between SV exo-endocytosis in synapses,^42^ our results provide a plausible explanation for the underlying biophysical principle, that is, the liquid-like nature of synapsin and various endocytic proteins assembled by EndoA1.

### Limitations of the study

The ability of EndoA1 full-length, as well as its isolated N-BAR domain, to undergo LLPS is compatible with a recent study by Mondal et al.,^25^ but is somewhat surprising, because it does not contain any intrinsically disordered regions (IDRs) which are believed to be prone to phase separate. Although mechanistic understanding of how the BAR domain undergoes LLPS is still lacking, the amphipathic nature of BAR-domains, commonly seen in BAR-domain-containing proteins, may provide the force to self-assemble, which eventually triggers condensate formation. Of interest, other N-BAR domains of Amph1 and BIN1 also phase separate *in vitro*.^25^ It will be interesting to see whether the capacity to undergo LLPS is a common feature of BAR domains.

Another limitation of this study is the lack of direct evidence to support a role of EndoA1 in formation and reassembly of SV clusters in presynaptic terminals. Further experiments involving EndoA1 loss of function and rescue with an EndoA1 mutant that specifically disables its capacity to undergo LLPS but retains full activity in SV endocytosis might help. However, as stated above, it is likely that the amphipathic property of the EndoA1-BAR domain may be responsible for its droplet formation, and if so, candidate mutations in the BAR domain will likely disrupt its membrane interaction and membrane deformation activity that are essential for SV endocytosis.^10^ Dissecting these two distinct roles included in the same domain, i.e., the potential to undergo LLPS and to mediate SV endocytosis, with rigorous biochemical as well as functional assays will be the key to determine the function of EndoA1 condensates in synaptic physiology.

## STAR★Methods

### Key resources table

**Table:** 

REAGENT or RESOURCE	SOURCE	IDENTIFIER
**Antibodies**		

Rabbit polyclonal anti-EndoI	A gift from Dr. Hans- Dieter Söling	#93
Guinea pig polyclonal anti-EndoI	Synaptic Systems	Cat#159 004 RRID: AB_2619854
Mouse monoclonal anti-synapsin 1	A gift from Dr. Reinhard Jahn	M10.22
Rabbit polyclonal anti-VGLUT1	Takamori et al.^52^	Shigeo3
Guinea pig polyclonal anti-VGLUT1	Synaptic Systems	Cat#135 304 RRID: AB_887878
Rabbit polyclonal anti-Complexin 1/2	Synaptic Systems	Cat#122 002 RRID: AB_887709
Mouse monoclonal anti-Bassoon	Enzo life science	Cat#SAP7F407 RRID: AB_2313990
Mouse monoclonal anti-Actin	Sigma	Cat#A5316 RRID: AB_476743
Mouse IgG HRP linked F(ab’) Fragment (sheep monoclonal)	Cytiva	Cat#GENA9310 RRID:AB_772193
Peroxidase AffiniPure Donkey Anti-Guinea Pig IgG (H+L)	Jackson ImmunoResearch	Cat# 706-035-148 RRID: AB_2340447
Alexa 488 goat anti-mouse IgG	Thermo Fisher Scientific	Cat#A11029 RRID: AB_2534088
Alexa 568 goat anti-rabbit IgG	Thermo Fisher Scientific	Cat#A11036 RRID: AB_10563566
AffiniPure Donkey Anti-Rabbit IgG (H+L)	Jackson ImmunoResearch	Cat#711-005-152 RRID: AB_2340585
AffiniPure Donkey Anti-Guinea Pig IgG (H+L)	Jackson ImmunoResearch	Cat#706-005-148 RRID:AB_2340443
AffiniPure Goat Anti-Mouse IgG, Fcγ subclass 1 specific	Jackson ImmunoResearch	Cat#115-005-205 RRID:AB_2338461
AffiniPure Goat Anti-Mouse IgG, Fcγ subclass 2a specific	Jackson ImmunoResearch	Cat#115-005-206 RRID:AB_2338462

**Bacterial and virus strains**		

Rosetta (DE3) pLysS Competent cells	Novagen	Cat#709563
f(Ub)-endophilin A1-EGFP-w	This paper	N/A (house making)
f(Ub)-EGFP-synapsin 1-w	This paper	N/A (house making)

**Chemicals, peptides, and recombinant proteins**		

Lipofectamine 2000 Transfection Reagent	Thermo Fisher Scientific	Cat#11668027
Hoechst 33342, Trihydrochloride, Trihydrate	Thermo Fisher Scientific	Cat#H1399
PEG8000	MP Biomedicals	Cat#194839
Cy3 Mono-Reactive Dye	Cytiva	Cat#23001
Cy5 Mono-Reactive Dye	Cytiva	Cat#25001
Alexa Fluor™ 647 NHS Ester	Thermo Fisher Scientific	Cat# A20006
Alexa Fluor™ 488 NHS Ester	Thermo Fisher Scientific	Cat# A20000
Alexa Fluor™ 405 NHS Ester	Thermo Fisher Scientific	Cat# A30000
1,6- Hexanediol	Sigma	Cat# 240117
Intersectin 1-(SH3)_5_ recombinant protein	This paper	N/A (house making)
Endophilin A1 recombinant protein	This paper	N/A (house making)
Endophilin A1-BAR recombinant protein	This paper	N/A (house making)

**Experimental models: Cell lines**		

Green monkey: COS7	RIKEN	N/A
Human: tsA201	A gift from Dr. Reinhard Jahn	N/A

**Recombinant DNA**		

pET28a(+)-HRV3C-intersectin 1-(SH3)_5_	This paper	NM_001110275.1
pGEX-endophilin A1	This paper	AF326561.1
pGEX-endophilin A1-BAR	This paper	AF326561.1
pEGFP-C1	Clontech	Cat#6084-1
pTagRFP-C1	Evrogen	Cat # FP141
pEGFP-intersectin 1-full length	A gift from Dr. Volker Haucke	NM_003024.3
pcDNA3.1-endophilin A1-TagRFP	This paper	AF326561.1
pcDNA3.1-endophilin A1-EGFP	This paper	AF326561.1
pTagRFP-dynamin 1	This paper	NM_001301737.1
pTagRFP-amphiphysin 1	This paper	NM_175007.2
piRFP670-amphiphysin 1	This paper	NM_175007.2
pTagRFP-synapsin 1	This paper	NM_013680.4
pEGFP-synapsin 1	This paper	NM_013680.4
pcDNA3.1-synaptophysin	This paper	NM_009305.2
pcDNA3.1-synaptophysin-EGFP	This paper	NM_009305.2
pCAG-kGP1	Hioki et al.^53^	N/A
pCAG4-RTR2	Hioki et al.^53^	N/A
pCAG-VSVG	Hioki et al.^53^	N/A
pFU(Endophilin A1-EGFP)W	This paper	AF326561.1
pFU(EGFP-Synapsin 1)W	This paper	NM_013680.4

**Software and algorithms**		

Fiji (ImageJ)	https://fiji.sc/#cite	RRID: SCR_002285
Figure J	https://imagej.net/FigureJ	N/A
Imaris version 9.6.0	Bitplane	RRID: SCR_007370
MS Excel 2016	Microsoft	RRID: SCR_016137
Python	https://anaconda.org	RRID: SCR_008394
Mathematica	https://www.wolfram.com/index.ja.html?source=footer	RRID: SCR_014448
Affinity Designer	https://affinity.serif.com/ja-jp/designer/	RRID: SCR_016952

### Resource availability

#### Lead contact

Further information and requests for resources should be directed to and will be fulfilled by the lead contact, Shigeo Takamori (stakamor@mail.doshisha.ac.jp).

#### Materials availability

All materials reported in this paper will be shared by the lead contact upon request.

### Experimental model and subject details

#### Animals

Female and male ICR mice (Japan SLC) at embryonic day 16 were used to prepare hippocampal cultures, except for 3D-STORM analysis. For 3D-STORM analysis, embryonic-day-21 Sprague-Dawley rats (Japan SLC) were used. Mice and rats were taken care at the animal house, and food and water could be obtained freely.

#### Bacterial strain

Rosetta (DE3) pLysS-competent cells were used for production of recombinant proteins.

#### COS7 and tsA201 cell culture

COS7 and tsA201 cells were grown in Dulbecco’s Modified Eagle’s Medium (DMEM) (High Glucose) (Wako) supplemented with 2 mM L-glutamine (Wako), 1 mM sodium pyruvate (Wako), 10% fetal bovine serum (FBS, Biosera) and 0.6% penicillin/streptomycin (Sigma) at 37°C in a humidified 5% CO_2_ incubator.

#### Neural cultures

Primary hippocampal cultures were prepared from embryonic-day-16 ICR mice or embryonic-day-21 Sprague-Dawley rats, as described previously,^32^^,^^54^ with slight modifications. Briefly, for experiments presented in Figures 8 and S6, hippocampi were dissected from embryonic-day-16 ICR mice and incubated with papain (90 units/mL, Worthington) for 40 min. Dissociated hippocampal cells were plated onto poly-D-lysine-coated coverslips at a cell density 20,000–30,000 cells/cm^2^. Neural cells were grown in neurobasal medium (Thermo Fisher Scientific) supplemented with 1.25% FBS, 2% B27 (Thermo Fisher Scientific), and 0.5 mM L-glutamine at 37°C, 5% CO_2_. 40 μM FUDR (Sigma) and 100 μM uridine (Sigma) were added to the culture medium to limit glial proliferation at 2–3 days *in vitro* (DIV). The growth medium was replaced with one-third fresh medium (neurobasal medium supplemented with 2% B27 and 0.5 mM L-glutamine) every 2–3 days until imaging experiments. For experiments presented in Figures 7 and S5, hippocampi were dissected from embryonic-day-21 Sprague-Dawley rats and incubated with 1% trypsin (Sigma) and 1 mg/mL DNase I (Sigma) for 5 min. Dissociated hippocampal cells were plated onto the glial cell layer precultured on poly-L-lysine- and laminin- (Thermo Fisher Scientific) coated coverslips at a cell density of 2,000 cells/cm^2^. Neural cells were grown in neurobasal medium (Thermo Fisher Scientific) supplemented with 2% B27 (Thermo Fisher Scientific), and 0.5 mM Glutamax (Thermo Fisher Scientific), 1 mM sodium pyruvate (Wako), and 1% penicillin/streptomycin (Thermo Fisher Scientific) at 37°C, 5% CO_2_. 2.5 μM Cytosine β-D-arabinofuranoside (Sigma) was added to the culture medium at 2 DIV to limit glial proliferation. About half of the growth medium was replaced with fresh medium once a week.

#### Study approval

All animal experiments in the S.T. laboratory at Doshisha University, and in the K.H. laboratory at the University of Tokyo, were carried out in accordance with respective institutional regulations for animal experiments based on the governmental Guideline for Proper Conduct of Animal Experiment and Related Activities, and were approved by the institutional committees of Doshisha University and the University of Tokyo.

### Method details

#### Molecular cloning

To construct each plasmid, corresponding coding sequences were amplified by polymerase chain reaction (PCR) using complementary DNAs reverse-transcribed from mouse brain total RNA or plasmids containing the corresponding cDNA as templates. For PCR, PrimeSTAR Max DNA Polymerase (Takara) was routinely used to amplify the desired DNA fragments, and the resulting PCR products were subcloned into appropriate vectors using In-Fusion (Takara) or using available restriction sites. Sequences of all plasmids were verified by DNA sequencing. Plasmids constructed in this paper are listed in the key resources table.

#### Protein expression and purification

6× His-ITSN1-(SH3)_5_, GST-Endophilin A1 and GST-Endophilin A1-BAR domain (corresponding to amino acids 1–247) were expressed in RosettaTM (DE3) pLysS-competent cells (Merck Millipore) in LB broth medium. Expression was induced by adding 1 mM isopropyl β-D-1- thiogalactopyranoside (IPTG) when the OD_600_ of the cell suspension reached 0.5–0.8. After incubation at 18°C for 16–18 h, suspension of bacterial cells was centrifuged at 1,482 × *g* for 30 min at 4°C in a JA-10 rotor (Beckman). The cell pellet was lysed in sonication buffer (for Endophilin A1: 20 mM Tris-HCl, 150 mM NaCl, 1 mM ethylenediaminetetraacetic acid (EDTA), 5 mM dithiothreitol (DTT), 1 mM phenylmethanesulfonyl fluoride (PMSF), pH 7.4; for Endophilin A1-BAR domain: 20 mM Tris- HCl, 300 mM NaCl, 1 mM EDTA, 5 mM DTT, 1 mM PMSF, pH 7.4; for 6× His-tagged proteins: 25 mM 2-[4-(2-Hydroxyethyl)-1-piperazinyl]ethanesulfonic acid (HEPES), pH 7.4) and centrifuged at 39,191 × *g* for 30 min at 4°C in a JA-20 rotor (Beckman). Supernatant was filtered through a 0.45 μm syringe filter and mixed with appropriate resin for purification at 4°C (for GST-tagged proteins, Glutathione Sepharose 4B (Cytiva); for 6× His-tagged proteins; TALON metal affinity resin (Clontech)). For initial screening to judge their potential to phase separate *in vitro* shown in Figures 1A and 1B, after washing (for GST-tagged protein: sonication buffer; for 6×His-tagged proteins: 25 mM HEPES, 5 mM imidazole, pH 7.4), each protein was eluted by elution buffer (for GST-tagged protein: 20 mM Tris-HCl, 150 mM NaCl, 1 mM EDTA, 20 mM Glutathione, pH 8.0; for 6× His-tagged proteins: 25 mM HEPES, 500 mM imidazole, pH 7.4). Eluted proteins were dialyzed (for GST-tagged protein: 25 mM HEPES, 150 mM NaCl, 1 mM DTT, pH 7.4; for 6× His-tagged proteins: 25 mM HEPES, 1 mM DTT, pH 7.4) using a dialysis membrane (Spectra/Por® Dialysis Membrane, MWCO: 20 kDa). For tag-free protein experiments in Figures 1C–1G, 2, and S2, after washing (for GST-tagged proteins: sonication buffer; for 6× His-tagged proteins; 25 mM HEPES, 5 mM imidazole, pH 7.4), affinity tags of each protein were cleaved with HRV-3C protease at 4°C overnight. Eluted proteins were then further purified using a BioLogic LP system (BioRad) implemented either with an ion-exchange column or a gel filtration column (for EndoA1 and EndoA1-BAR, UNO® Monolith Anion Exchange Columns and a Superdex 200 pg 16/600 column (GE Healthcare); for ITSN1-(SH3)_5_, a Superdex 200 pg 16/600 column (GE Healthcare)). Purified proteins were snap-frozen in liquid nitrogen and stored at −80°C until use. Protein concentrations were determined with a PierceTM BCA Protein Assay Kit (Thermo Fisher Scientific) and purity was confirmed with SDS-PAGE and Coomassie brilliant blue (CBB) staining.

#### Protein labeling with fluorophores

Purified proteins were labeled with Cy3 or Cy5 maleimide mono-reactive dyes (Cytiva) in protein solution buffer at a concentration ratio of 1 mg protein/0.05 vial by incubating for 30 min at RT with gentle agitation every 10 min. Fluorophores and other small molecules were removed from proteins by passing them over a PD-10 desalting column (Cytiva) and protein concentrations were adjusted with an Amicon Ultra cartridge (0.5 mL, 10 kDa cut-off, Merck Millipore). Labeling efficiency of all proteins was ∼15%, calculated with a BioPhotometer Plus (Eppendorf). Protein concentrations of final samples were determined by SDS-PAGE and CBB staining.

#### LLPS assay *in vitro*

LLPS was induced by mixing a solution containing recombinant proteins with an induction buffer (150 mM NaCl, 25 mM HEPES, pH 7.4, ± 0–10% (w/v) PEG8000) at the desired concentrations. The mixture was put in a 27-mm glass-bottomed dish (Fine Plus International Ltd) or 24 × 50 mm cover-glass (Matsunami), covered with a cover-glass and the gap between the glass-bottomed dish and the cover-glass was sealed with grease (DOW CORNING TORAY) to prevent evaporation, and observed with a DIC, a Carl Zeiss LSM-900 confocal fluorescence microscope with a 63× oil-immersion (NA = 1.40) objective lens or an Olympus FV-1000 confocal fluorescence microscope with a 60× oil-immersion (NA = 1.42) objective lens. Line scan analysis was performed using Fiji. To quantify fluorescence, a straight line (Line width = one pixel) was drawn across a droplet. Intensity of the line was calculated using a plot profile tool in Fiji. Intensities were normalized against the highest intensities within the selected areas.

#### Transient transfection in COS7 cells

Cell cultures (COS7) were plated either onto poly-D-lysine (PDL) coated coverslips (Matsunami MicroCover Glass, thickness = No. 0) framed in a Nunc™ 4 well dish (for microscopy analyses) or PDL-coated Falcon® 6-well Clear Flat Bottom TC-treated Multiwell Cell Culture Plate (for cell lysate preparations) and transfected with plasmid DNAs (each 0.25 μg DNA) using Lipofectamine 2000 Transfection Reagent (Thermo Fisher Scientific). All experiments were performed 2 days after transfection.

#### Acquisition of live heterologous cell images

Live cell fluorescent images of COS7 cells were taken with a Carl Zeiss LSM-900 confocal fluorescence microscope with 63× oil-immersion (NA = 1.40) objective lens at RT. Imaging was conducted in artificial cerebrospinal fluid (ACSF; 140 mM NaCl, 2.4 mM KCl, 10 mM HEPES, 10 mM D-Glucose, 2 mM CaCl_2_, 1 mM MgCl_2_, pH 7.4). Images were acquired under the control of Zeiss efficient navigation (ZEN) software. Image processing was performed using the image analysis software, Fiji^55^ with the FigureJ plug-in.^56^

#### Fluorescence recovery after photobleaching (FRAP)

FRAP (fluorescence recovery after photobleaching) experiments *in vitro* and in COS7 cells were performed using a Carl Zeiss LSM-900 confocal fluorescence microscope with a 63× oil-immersion (NA = 1.40) objective lens at RT. According to the size of droplets, regions of interest (ROI) were chosen. Droplets were bleached using a 488-nm, 561-nm, or 633 nm laser (Setting of laser power: 100%). The fluorescence intensity of ROI was monitored at a frame interval of 5 s. Acquired fluorescence images were analyzed using Fiji software. Images were first background-corrected using a ‘rolling ball’ function with a radius of 50 pixels (http://fiji.sc/Rolling_Ball_Background_Subtraction). After correcting the lateral movement of droplets with the Stack Reg plugin (http://bigwww.epfl.ch/thevenaz/stackreg/), a circular region of interest (ROI) was positioned at the center of the droplet. Change of mean fluorescence in the ROI was obtained with the Time Series Analyzer 3 plugin (https://imagej.nih.gov/ij/plugins/time-series.html). Data were normalized to pre- and post-bleaching points and shown as means ± standard errors of the means (s.e.m).

#### Validation of transfection efficiency in COS7 cells

To evaluate transfection efficiency, COS7 cells were transfected with EndoA1-EGFP (0.25 μg) using Lipofectamine 2000 Transfection Reagent (Thermo Fisher Scientific). After 2 days, cells were incubated with Hoechst 33342 (1:2,000) in phosphate-buffered saline (PBS) for 10 min at 37°C. After incubation, cells were visualized with an Olympus IX-71 inverted microscope equipped with a 60× (NA = 1.35) oil-immersion objective lens and 75W xenon arc lamp (Ushio). Transfection efficiency was manually calculated as follows: GFP positive cells/Hoechst positive cells. Hoechst was imaged with 365/5 nm excitation and 440/20 nm emission filters. EGFP was imaged with 470/22 nm excitation and 514/30 nm emission filters.

#### Preparation of cell lysates

At 14 days *in vitro* (DIV), cultured neurons derived from hippocampi of ICR mice were used to prepare neural cell lysates. COS7 cells were grown to approximately 80–90% confluency and were used to prepare the cell lysates. Cells were scraped with radioimmunoprecipitation (RIPA) buffer (50 mM Tris-HCl pH 8.0, 150 mM NaCl, 0.5% (w/v) sodium deoxycholate, 0.1% SDS, 1% NP40) and centrifuged at 20,000 × g for 30 min at 4°C. Supernatants were collected and protein concentrations were determined with a PierceTM BCA Protein Assay Kit (Thermo Fisher Scientific). Samples were mixed with 2× SDS sample buffer supplemented with 40 mM dithiothreitol (DTT), and were boiled for 3 min at 95°C.

#### Western blotting

Cell lysates were electrophoresed on 10% SDS polyacrylamide gels and transferred to PVDF membranes (Millipore) for 30 min with a semi-dry blotting device (BioRad). Membranes were blocked for 30 min at RT with blocking buffer (5% skim milk in Tris-buffered saline (pH 7.4) supplemented with 0.05% Tween 20 (TBS-T)). Primary antibodies were used at the following dilutions: anti-EndoI (1:1,000), and anti-β-actin (1:1,000). Secondary antibodies conjugated with HRP were used at 1:10,000. Chemiluminescence signals of the HRP enzymatic reaction were detected with Western Lightning Plus-ECL (PerkinElmer) using a Molecular Imager ChemiDoc (BioRad). Signal intensities of bands were quantified using a Gel tool in Fiji software.

#### Droplet detection in COS7 cells

Droplets in COS7 cells were analyzed using Fiji software. Co-localization analysis was performed using Fiji software. Images were first background-corrected using a ‘rolling ball’ function with a radius of 50 pixels (http://fiji.sc/Rolling_Ball_Background_Subtraction). Images in heterologous cells were smoothed with a Gaussian spatial filter (σ = 1.00) and binarized. Co-localization rates were calculated using Plugin, JACoP.

#### Immunocytochemistry of COS7 cells

Immunocytochemistry of COS7 cells was performed 2 days after transfecting plasmids. COS7 cells were fixed with 4% (w/v) paraformaldehyde (PFA) and 4% (w/v) sucrose in PBS for 15 min at RT and then permeabilized with 0.2% Triton X-100 in PBS for 10 min at RT. After blocking with PBS containing 0.3% bovine serum albumin (BSA, Sigma), cells were incubated with primary antibody containing 0.3% BSA for 60 min at RT. Primary antibodies were used at the following dilution: anti-synaptophysin (1:1,000). After washing with 0.3% BSA in PBS, cells were incubated with secondary antibody containing 0.3% BSA for 60 min at RT. Secondary antibodies conjugated with fluorophore were used at 1:1,000. Finally, after extensive washing, cells were visualized with a Carl Zeiss LSM-900 confocal fluorescence microscope with a 63× oil-immersion (NA =1.40) objective lens. Acquired images were analyzed with Fiji.

#### Correlative light and electron microscopy

Correlative light and electron microscopy was conducted as previously described.^57^ COS7 cells expressing either EndoA1-EGFP and TagRFP-synapsin 1, or EndoA1-EGFP, TagRFP-synapsin 1, and Synaptophysin (Syph) were cultured in DMEM supplemented with 10% FBS and 1% penicillin/streptomycin on custom-made, gridded coverslip-bottom dishes (based on IWAKI 3922-035; coverslips were affixed with inverse orientation), pre-coated with carbon by vacuum evaporation and subsequently coated with poly-D-lysine (Sigma-Aldrich). Live cell imaging was performed using Nikon A1R confocal microscopy with a 100x oil-immersion (NA = 1.45) objective lens at 37°C under a 5% CO_2_ atmosphere. After acquisition of fluorescent images using confocal microscopy (Nikon Ti2 Eclipse A1R, Nikon), cells were immediately fixed with 2% paraformaldehyde (Electron Microscopy Sciences) and 0.5% glutaraldehyde (Electron Microscopy Sciences) in 0.05 M phosphate buffer (0.01 M sodium dihydrogenphosphate dihydrate, 0.04 M disodium hydrogenphosphate; pH 7.4) at 4°C for 1 h. Following washing with 0.1 M phosphate buffer, cells were then fixed with 2.5% glutaraldehyde in 0.1 M phosphate buffer (pH 7.4) for 24 h at 4°C. After washing with 0.1 M phosphate buffer, cells were post-fixed with 1% (w/v) OsO4 (Electron Microscopy Sciences), 1.5% (w/v) potassium ferrocyanide (Fujifilm Wako Pure Chemical Corporation) in a 0.05 M phosphate buffer for 30 min. After being rinsed 3× with H_2_O, cells were stained with 1% (w/v) thiocarbohydrazide (Sigma-Aldrich) for 5 min. After rinsing 3× with H_2_O cells were stained with 1% OsO4 in H_2_O for 30 min. After rinsing 2× with H_2_O at RT and 3× with H_2_O at 50°C, cells were treated with Walton’s lead aspartate (0.635% (w/v) lead nitrate (Sigma-Aldrich) and 0.4% (w/v) aspartic acid-KOH (pH 5.2, Sigma-Aldrich)) at 50°C for 20 min. Cells were dehydrated with an ascending ethanol series (10 min each in 50% on ice, 70% on ice, 10 min in 90% at RT, and 5 min in 95% ethanol/H_2_O and 5 min in 100% ethanol 3× at RT). Dehydrated cells were embedded in epoxy resin (Epok812, Oken) by overlaying gridded glass with resin-filled beam capsules (TAAB). Polymerization was carried out at 42°C for 12 h and 60°C for 72 h. After polymerization, the gridded coverslip was removed and the resin block was trimmed to about 500 μm × 500 μm. The block was sectioned using an ultramicrotome (EM UC7, Leica) equipped with a diamond knife (Ultra JUMBO 45 degree, DiATOME) to cut 40 nm-thick sections. These ultra-thin sections were collected on cleaned silicon wafer strips and imaged with a scanning electron microscope (JSM-IT800, JEOL). Imaging at high magnification was done at 2 keV accelerating voltage, 5 keV specimen voltage, 200 pA beam current, 2,560 × 1,920 frame size, 6 mm working distance, 2.56 × 1.92 μm field of view and 14.1 μs dwell time, using a Scintillator Backscattered Electron Detector in Beam Deceleration mode. The final pixel size was 1 nm square. Image processing, correlation of light and electron microscopic images, and 3D visualization was performed using Fiji and Imaris version 9.6.0 (Bitplane).

#### Immunocytochemistry of neural cultures

Immunocytochemistry of neural cultures was performed at 14–22 days *in vitro* (DIV). Neural cultures were fixed with 4% (w/v) PFA and 4% (w/v) sucrose in PBS for 15 min at RT and then permeabilized with 0.2% Triton X-100 in PBS for 10 min at RT. After blocking with PBS containing 0.3% BSA, cells were incubated with primary antibody containing 0.3% BSA for 60 min at RT. Primary antibodies were used at the following dilutions: anti-synapsin1 (1:1,000), anti-EndoI (1:1,000), anti-Bassoon (1:2,000), anti-Complexin 1/2 (1:500) and anti-VGLUT1 (1:1,000 for Shigeo3 and 1:2,000 for Synaptic Systems). After washing with 0.3% BSA in PBS, cells were incubated with secondary antibody containing 0.3% BSA for 60 min at RT. Secondary antibodies conjugated with fluorophore were used at 1:1,000. Finally, after extensive washing, cells were post-fixed with 4% PFA for 15 min at RT and mounted in Prolong Diamond. Specimens were visualized with Carl Zeiss LSM-900 and Leica TCS SP8 confocal fluorescence microscopes with a 63× oil-immersion (NA =1.40) and a 100× oil-immersion (NA = 1.40) objective lens, respectively. Acquired images were analyzed with Fiji and images shown in figures (Figures 7, 8A, 8B, S5, and S6A) were then smoothed with a Gaussian spatial filter (the radius of 1 pixel (200 nm) for Figures 7 and S5; σ = 1.00 for Figures 8A, 8B, and S6A).

#### Analysis of the dispersion of presynaptic molecules

Neural cultures were exposed to 3% 1,6-hexanediol (HD) in normal ACSF (140 mM NaCl, 2.4 mM KCl, 10 mM HEPES, 10 mM D-glucose, 2 mM CaCl_2_, 1 mM MgCl_2_, pH 7.4) or high K^+^ buffer (97.4 mM NaCl, 45 mM KCl, 10 mM HEPES, 10 mM D-glucose, 2 mM CaCl_2_, 1 mM MgCl_2_, pH 7.4) for 1 min and immediately fixed. In recovery experiment, neurons were exposed to high K^+^ buffer for 1 min. Then medium was replaced with normal ACSF and neurons were incubated for 10 min and fixed. In Ca^2+^-free experiment, neurons were exposed high K^+^ and Ca^2+^-free buffer (97.4 mM NaCl, 45 mM KCl, 10 mM HEPES, 10 mM D-glucose, 0 mM CaCl_2_, 1 mM MgCl_2_, 10 mM EDTA, pH 7.4) for 1 min and immediately fixed. Fixed neurons were immunostained with the normal procedure, described above (refer to immunocytochemistry of neural cultures).

To evaluate the degrees of diffusion for presynaptic molecules along the axons, coefficient of variation (CV) analysis was performed using Fiji software. First, acquired images were background-corrected using a ‘rolling ball’ function with a radius of 50 pixels (http://fiji.sc/Rolling_Ball_Background_Subtraction). Images were then smoothed with a Gaussian spatial filter (σ = 1.00). Finally, line (5 pixels) scans of axon-like areas were performed, and the signal variability and average luminance were calculated. The CV is expressed by the following equation: signal variability/average luminance.

#### STORM imaging data acquisition

For two-color 3D-STORM imaging, specimens were immunostained with secondary antibodies bearing reporter-activator dyes (Alexa Fluor 647-488 and Alexa Fluor 647-405). FluoSpheresTM Carboxylate-Modified Microspheres (F-8810, Thermo Fisher Scientific, 1:7,500) were bound to specimens in PBS supplemented with 50 mM MgCl_2_ for 30 min and used as fiducial markers. Specimens were then mounted in STORM buffer (50 mM HEPES (pH 8.0), 10 mM NaCl, 10% glucose, 60% sucrose, 2% 2-mercaptoethanol, 0.5 mg/mL glucose oxidase and 0.04 mg/mL catalase). STORM imaging was performed on a custom-built microscope with a 100× oil-immersion (NA =1.40) objective lens, a piezo-positioning stage (P-733.3, PI), and a custom-made objective holder that minimized mechanical sample drift during experiments^58^. A 640-nm laser beam (OBIS, Coherent) passed through a Cy5 excitation filter (Semrock) was used at an excitation intensity of ∼7.5 kW/cm^2^ to excite and turn off Alexa Fluor 647 molecules, enabling single-molecule imaging. Laser beams of 405 nm and 488 nm (OBIS, Coherent) were used to re-activate Alexa Fluor 647 molecules via excitation of Alexa Fluor 405 and Alexa Fluor 488 molecules, respectively. A multi-band dichroic mirror (Semrock) and a bandpass emission filter (710QM80, Omega) were used to collect Alexa Fluor 647 fluorescence. For 3D astigmatism-based calibration, a cylindrical lens was inserted into the detection path.

Raw images of 128 × 128-pixel resolution (image pixel size of 200 nm) were acquired with an EMCCD camera (iXon3 860, Andor) at a 100-Hz sampling rate as 16-bit tiff image stacks. 10,000 imaging frames were alternately acquired with 488-nm and 405-nm laser excitation for a total of 120,000 imaging frames (60,000 imaging frames for each channel) for a single image reconstruction. Excitation intensities of the 488-nm and 405-nm lasers were gradually increased during recording from 0.25 to 8 W/cm^2^ and from 1 to 32 W/cm^2^, respectively, so that fluorescent signals from activated molecules did not spatially overlap. In-focus FluoSpheres, which appear as constant fluorescent spot signals, were included in imaging fields for correction of sample drift.

Reconstruction of sub-diffraction-limit images from raw images was performed using custom-written software based on single-molecule localization with least-square 2D Gaussian fitting. Briefly, raw images were smoothed with a 2D Gaussian filter with a radius of 1 pixel (200 nm). Lateral localizations (x and y coordinates) of individual Alexa Fluor 647 molecules were determined by estimating center positions of fluorescent signals with least-square fitting of the 2D Gaussian model. Axial localizations (z coordinates) were determined using an astigmatism-based calibration method, in which differences in values of width parameters in x and y directions for 2D Gaussian fitting were used. Sample drift during recording was corrected using fluorescent fiducial markers. 3D-STORM images were then reconstructed by pixelating Alexa Fluor 647 localization (x, y, and z coordinates) datasets with 10-nm binning for each color channel (488-nm laser activated and 405 nm laser activated series).

For evaluation of nanoscale co-localization of synaptic proteins, synapse structures in two-color 3D-STORM images were manually and randomly selected. Selected images were smoothed with a 3D Gaussian filter with a radius of 2 voxels (20 nm), and then binarized with the Otsu algorithm. Positive voxels were divided into connected components, and each component was then labeled as an individual cluster. The largest cluster in each synapse for each synaptic protein was extracted for further analysis while all smaller clusters were excluded from analysis. The center-of-mass of each protein cluster was quantified by calculating mean voxel coordinates. The volume of each cluster was quantified by counting total voxels of the extracted major cluster for each synaptic protein. Manders coefficients were quantified by calculating the ratio of voxel overlap (both positive) to total voxels of the reference protein cluster. Image data analysis was performed using Mathematica (Wolfram).

#### Live cell imaging of neural cultures

To monitor behaviors of EndoA1 and synapsin in response to electrical stimulation in neurons, neuronal cultures were transduced with lentiviral vectors encoding either EndoA1-EGFP or EGFP-synapsin 1 at 7 DIV. Lentiviral vectors were produced using tsA201 cells as hosts, as described previously.^54^ Briefly, tsA201 cells were plated on 100-mm TC-treated cell culture dish (CORNING). After 2–3 h, cells were transfected with 17 μg of lentiviral backbone vector based on pFUGW and helper plasmids (pCAG-kGP1 10 μg, pCAG4-RTR2 5 μg and pCAG-VSVG 5 μg) by calcium phosphate transfection.^59^ After 16 h, cultured medium was replaced with fresh neural culture medium. After another 48 h, supernatants were collected, centrifuged at 500 × *g* for 15 min, and were filtered through a 0.45 μm filter (Millipore). Viral aliquots were flash frozen in liquid nitrogen and stored at −80°C until use.

Live cell imaging was carried out on an Olympus IX-71 inverted microscope equipped with a 60× (NA = 1.35) oil-immersion objective lens and 75-W xenon arc lamp (Ushio). Cultured neurons (14–22 DIV) on glass coverslips were placed in a custom-made imaging chamber with continuous perfusion of artificial cerebrospinal fluid (ACSF; 140 mM NaCl, 2.4 mM KCl, 10 mM HEPES, 10 mM D-Glucose, 2 mM CaCl_2_, 1 mM MgCl_2_, pH 7.4) at RT. Raw images of 512 × 512-pixel resolution were acquired with an ORCA-Flash 4.0 sCMOS camera (Hamamatsu Photonics) at a 0.2 Hz sampling rate as 16-bit tiff image stacks. EGFP fluorescence was imaged with 470/22 nm excitation and 514/30 nm emission filters. Note that in the case of electrical stimulation experiments, the imaging solution was set to ACSF with 0.02 mM CNQX, and 0.025 mM D-APV. Electrical stimulation was performed with custom-made bipolar platinum electrodes 2.0 mm apart for 1-ms constant current pulses (10 mA) controlled by pCLAMP Software (Molecular Devices).

Image analysis was performed with Fiji software^55^ using the Time Series Analyzer plugin. Acquired images were background subtracted using a rolling ball algorithm with a radius of 50 pixels. Image drift was corrected using the Fiji plugin (correct 3D drift tool). Circular regions of interest (ROIs, diameter = 1.08 μm) were manually positioned at the center of punctate fluorescence signals. EGFP fluorescence in ROIs was calculated as ΔF (F – F_t = 15_)/F0. ΔF (F – F_t = 15_)/F0 of ∼10 boutons from a single experiment (512 × 512 pixels) were averaged and expressed as n = 1. The mobile fraction is the absolute value of the ΔF (F – F_t = 15_)/F0 at t = 75 of each experiment. To calculate the time constant of dispersion, the ΔF (F – F_t = 15_)/F0 at t = 15–75 of each experiment was fitted with monoexponentially decay curve [y = A (1 – *e*^−t/τ^)] using Solver, Excel add-in program. To calculate the time constant of recovery, the ΔF (F – F_t = 75_)/F0 at t = 75–320 of each experiment was fitted with a monoexponential decay curve [y = A (1 – *e*^−t/τ^)] using Solver, an Excel add-in program. Note that a time constant of dispersion or recovery that did not fit within the imaging time was excluded from all data analysis.

#### Study design

We confirmed that all experimental findings were reliably reproduced. Randomization and blinding were not used in this study. Data exclusions were clearly indicated with the exclusion criteria of the data in the relevant section. Sample size was determined based on the previous publications that included similar research methods (examples; Milovanovic et al.,^4^ Park et al.,^6^ and Lopez-Hernandez et al.^54^)

### Quantification and statistical analysis

Data were analyzed with Excel 2016 and Python. All data are given as means ± standard errors of the means (s.e.m). All statistical analyses were two-tailed, and levels of statistical significance are indicated by asterisks: ∗p < 0.05, ∗∗p < 0.01, ∗∗∗p < 0.001. n.s.; not significant.

## Data Availability

• All data reported in this paper will be shared by the lead contact upon request. • This paper does not report original code. • Any additional information required to reanalyze the data reported in this paper is available from the lead contact upon request.
