# Supplementary material for: Compartmentalization of soluble endocytic proteins in synaptic vesicle clusters by phase separation
Source: iScience. 2023 May 6;26(6):106826. doi: 10.1016/j.isci.2023.106826 (PMC10209458; doi:10.1016/j.isci.2023.106826)
Supplement: Document S1. Figures S1–S6 [file mmc1.pdf]

iScience, Volume *26*

Supplemental information

Compartmentalization of soluble endocytic proteins in synaptic vesicle clusters by phase separation

Tomofumi Yoshida, Koh-ichiro Takenaka, Hirokazu Sakamoto, Yusuke Kojima, Takumi Sakano, Koyo Shibayama, Koki Nakamura, Kyoko Hanawa-Suetsugu, Yasunori Mori, Yusuke Hirabayashi, Kenzo Hirose, and Shigeo Takamori

SUPPLEMENTAL FIGURES

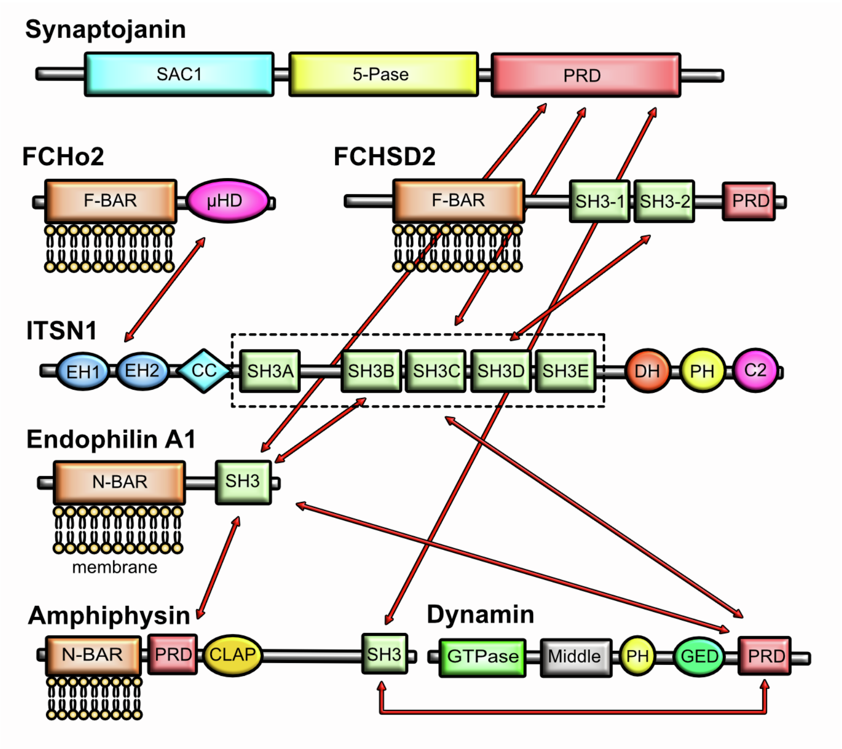

Figure S1. Various endocytic proteins interact to form multivalent protein networks, related to Figure 1

Domain structures of major endocytic proteins related to our study (see ref. [S1] for more detail). Synaptojanin consists of 1,575 amino acids (aa) in the human sequence, and has three characteristic domains, the SAC1, 5-phosphatase (5-Pase), and proline-rich domains (PRD). FCHo2 consists of 376 aa and has two characteristic domains, the F-BAR and μHD domains. FCHSD2 consists of 740 aa and has four characteristic domains, the F-BAR domain, two Src homology 3 (SH3) domains and PRD. Intersectin 1 (ITSN1) consists of 1,721 aa and has various characteristic domains, two N-terminal Eps15 homology domains (EH1/2), a coiled-coil domain (CC), and a tandem repeat of five SH3 domains (designated as SH3A-SH3E). The long splice variant is extended by a C-terminal Dbl homology domain (DH), a pleckstrin homology domain (PH), and a Ca^{2+}-binding domain (C2), which lack the short splicing variant of ITSN1. Endophilin A1 (EndoA1) consists of 352 aa and has two characteristic domains, the N-BAR and SH3 domains. Amphiphysin (Amph) consists of 695 aa and has four characteristic domains, the N-BAR, PRD, Clathrin-AP2-binding (CLAP) and SH3 domains. Dynamin consists of 866 aa and has five characteristic domains, including the GTPase, Middle, PH, GED and PRD domains. Red arrows indicate putative protein-protein interactions reported in the literature [S2-S8]. Note that both the N- and F-BAR domains have been demonstrated to bind lipid membranes *in vitro* [S9].

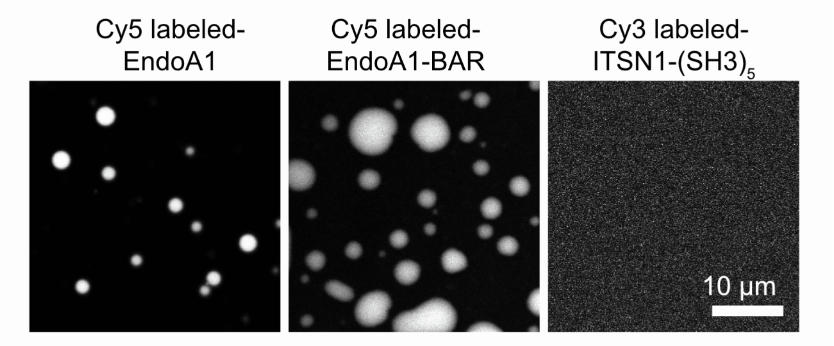

Figure S2. Representative images of a phase separation assay of chemical fluorophore-labeled proteins, related to Figure 2

The image shows Cy5-labeled EndoA1 (20 μM) (left) and Cy5-labeled EndoA1-BAR (middle) (20 μM) undergo LLPS in the presence of 10% PEG. In contrast, Cy3-labeled ITSN1-$(SH3)_5$ (20 μM) does not undergo LLPS (right).

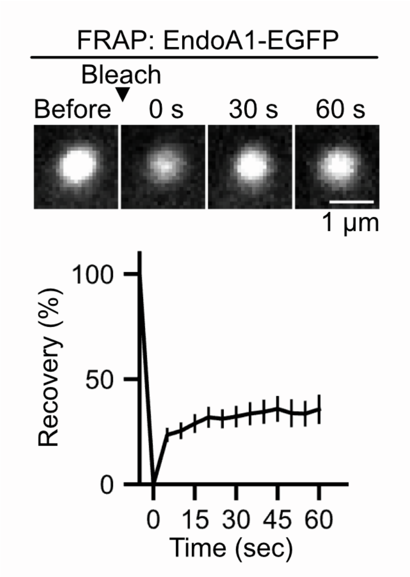

Figure S3. FRAP of EndoA1-EGFP condensates in COS7 cells, related to Figure 3

Fluorescence recovery after photobleaching (FRAP) of EndoA1-EGFP droplets in COS7 cells. Images were taken at 0.2 Hz. The trace at the bottom is the mean ± s.e.m of 16 droplets observed in multiple COS7 cells.

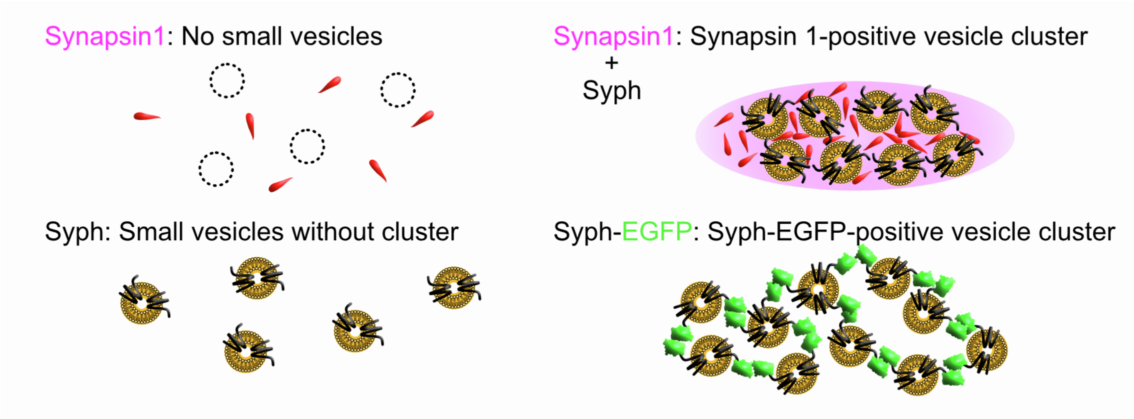

Figure S4. Schematic diagram of experimental reconstruction of SV-like clusters in COS7 cells, related to Figure 4

Previous studies have demonstrated that expression of synapsin 1 does not result in condensate formation (upper left), whereas expression of tag-free synaptophysin (Syph) promotes the generation of small vesicles in fibroblast cells (lower left) [S10, S11]. Notably, when both synapsin and tag-free Syph are co-expressed, synaptic vesicle-like clusters wrapped in synapsin 1-condensates emerge in the cytoplasm (upper right). Furthermore, mere expression of Syph C-terminally tagged with EGFP (Syph-EGFP) also produced SV-like clusters irrespective of synapsin 1 (lower right).

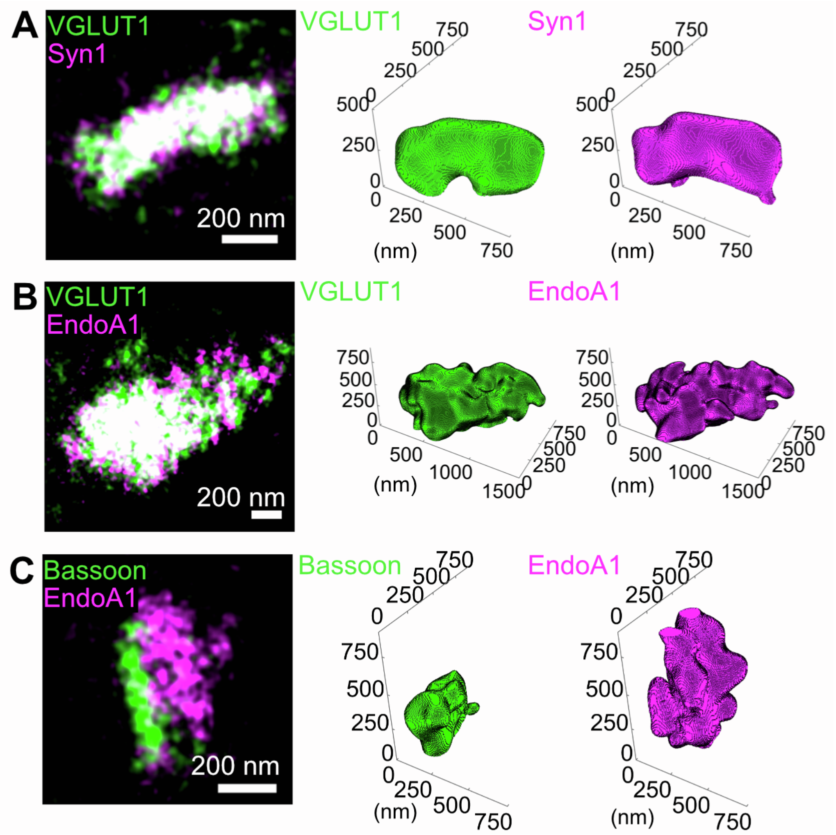

Figure S5. Representative STORM images of synaptic molecules, related to Figure 7 (A) A representative 2D-projected two-color STORM image of VGLUT1 (green) and synapsin 1 (Syn1) (magenta) at a hippocampal synapse. Right panels show 3D visualization of major clusters of VGLUT1 and Syn1 molecules, respectively, constructed from the image at left. (B) A representative 2D-projected two-color STORM image of VGLUT1 (green) and EndoA1 (magenta) at a hippocampal synapse. Right panels show 3D visualization of major clusters of VGLUT1 and EndoA1 molecules, respectively, constructed from the left image. (C) A representative 2D-projected two-color STORM image of bassoon (green) and EndoA1 (magenta) at a hippocampal synapse. Right panels show 3D visualization of major clusters of bassoon and EndoA1 molecules, respectively, constructed from the left image.

Scale bars = 200 nm

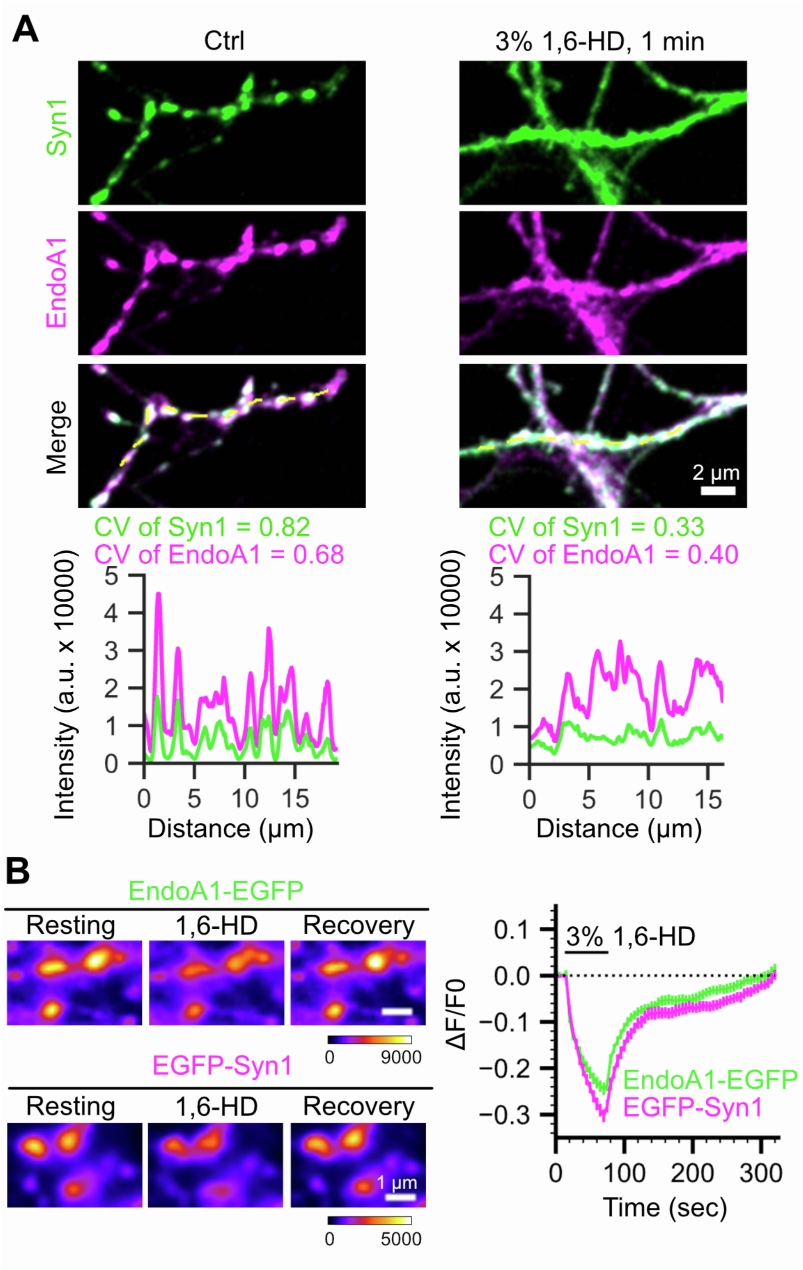

Figure S6. Characterization of EndoA1 condensates in hippocampal neurons, related to Figure 8 (A) Examples of coefficient of variation (CV) analysis of presynaptic molecules. Images show immunolabeling of synapsin 1 (Syn1, green) and EndoA1 (magenta) in hippocampal neurons under control conditions (left) or 1 min after 1,6-HD treatment (right). Yellow dotted lines indicate the area of line-scan analysis. Bottom traces show results of the line-scan analysis and representative CV values (arbitrary units, a.u.) measured from the images shown above. Scale bar = 2 μm. (B) EndoA1 and Syn1 have liquid-like properties in presynaptic terminals. Live fluorescence imaging of EndoA1-EGFP or EGFP-Syn1 in hippocampal neural cultures (left panel). These pseudo-color images show that punctate signals of EndoA1-EGFP or EGFP-Syn1

disperse upon application of 3% 1,6-hexanediol (1,6-HD) and recover after washout (Resting; start point of the live fluorescence imaging, 1,6-HD; the endpoint of 1,6-HD application, Recovery; the endpoint of live fluorescence imaging). Horizontal pseudo-color scales show fluorescence intensity values of EGFP (arbitrary units, a.u.). Scale bar = 1 μm. The time course of EndoA1-EGFP or EGFP-Syn1 fluorescence in punctate structures during 1,6-HD application (right panel). Traces represent the mean of $\Delta F/F_0$ within presynaptic-like structures ± s.e.m with n = 25 images and n = 22 images for EndoA1-EGFP and EGFP-Syn1, respectively. Data were collected at 0.2 Hz under an epifluorescence microscope.

SUPPLEMENTAL REFERENCES

S1. McMahon, H.T., and Boucrot, E. (2011). Molecular mechanism and physiological functions of clathrin-mediated endocytosis. Nat Rev Mol Cell Biol *12*, 517-533. 10.1038/nrm3151.

S2. Almeida-Souza, L., Frank, R.A.W., Garcia-Nafria, J., Colussi, A., Gunawardana, N., Johnson, C.M., Yu, M., Howard, G., Andrews, B., Vallis, Y., and McMahon, H.T. (2018). A Flat BAR Protein Promotes Actin Polymerization at the Base of Clathrin-Coated Pits. Cell *174*, 325-337.e314. 10.1016/j.cell.2018.05.020.

S3. David, C., McPherson, P.S., Mundigl, O., and de Camilli, P. (1996). A role of amphiphysin in synaptic vesicle endocytosis suggested by its binding to dynamin in nerve terminals. Proc Natl Acad Sci U S A *93*, 331-335. 10.1073/pnas.93.1.331.

S4. Henne, W.M., Boucrot, E., Meinecke, M., Evergren, E., Vallis, Y., Mittal, R., and McMahon, H.T. (2010). FCHo proteins are nucleators of clathrin-mediated endocytosis. Science *328*, 1281-1284. 10.1126/science.1188462.

S5. Micheva, K.D., Ramjaun, A.R., Kay, B.K., and McPherson, P.S. (1997). SH3 domain-dependent interactions of endophilin with amphiphysin. FEBS Lett *414*, 308-312. 10.1016/s0014-5793(97)01016-8.

S6. Pechstein, A., Gerth, F., Milosevic, I., Japel, M., Eichhorn-Grunig, M., Vorontsova, O., Bacetic, J., Maritzen, T., Shupliakov, O., Freund, C., and Haucke, V. (2015). Vesicle uncoating regulated by SH3-SH3 domain-mediated complex formation between endophilin and intersectin at synapses. EMBO Rep *16*, 232-239. 10.15252/embr.201439260.

S7. Ringstad, N., Nemoto, Y., and De Camilli, P. (1997). The SH3p4/Sh3p8/SH3p13 protein family: binding partners for synaptojanin and dynamin via a Grb2-like Src homology 3 domain. Proc Natl Acad Sci U S A *94*, 8569-8574. 10.1073/pnas.94.16.8569.

S8. Yamabhai, M., Hoffman, N.G., Hardison, N.L., McPherson, P.S., Castagnoli, L., Cesareni, G., and Kay, B.K. (1998). Intersectin, a novel adaptor protein with two Eps15 homology and five Src homology 3 domains. J Biol Chem *273*, 31401-31407. 10.1074/jbc.273.47.31401.

S9. Masuda, M., and Mochizuki, N. (2010). Structural characteristics of BAR domain superfamily to sculpt the membrane. Semin Cell Dev Biol *21*, 391-398. 10.1016/j.semcdb.2010.01.010.

S10. Johnston, P.A., Cameron, P.L., Stukenbrok, H., Jahn, R., De Camilli, P., and Sudhof, T.C. (1989). Synaptophysin is targeted to similar microvesicles in CHO and PC12 cells. EMBO J *8*, 2863-2872. 10.1002/j.1460-2075.1989.tb08434.x.

S11. Park, D., Wu, Y., Lee, S.E., Kim, G., Jeong, S., Milovanovic, D., De Camilli, P., and Chang, S. (2021). Cooperative function of synaptophysin and synapsin in the generation of synaptic vesicle-like clusters in non-neuronal cells. Nat Commun *12*, 263. 10.1038/s41467-020-20462-z.
